# Regulation of PERK Signaling and Leukemic Cell Survival by a Novel Cytosolic Isoform of the UPR Regulator GRP78/BiP

**DOI:** 10.1371/journal.pone.0006868

**Published:** 2009-08-31

**Authors:** Min Ni, Hui Zhou, Shiuan Wey, Peter Baumeister, Amy S. Lee

**Affiliations:** Department of Biochemistry and Molecular Biology and the USC/Norris Comprehensive Cancer Center, University of Southern California Keck School of Medicine, Los Angeles, California, United States of America; Texas A&M University, United States of America

## Abstract

The unfolded protein response (UPR) is an evolutionarily conserved mechanism to allow cells to adapt to stress targeting the endoplasmic reticulum (ER). Induction of ER chaperone GRP78/BiP increases protein folding capacity; as such it represents a major survival arm of UPR. Considering the central importance of the UPR in regulating cell survival and death, evidence is emerging that cells evolve feedback regulatory pathways to modulate the key UPR executors, however, the precise mechanisms remain to be elucidated. Here, we report the fortuitous discovery of GRP78va, a novel isoform of GRP78 generated by alternative splicing (retention of intron 1) and alternative translation initiation. Bioinformatic and biochemical analyses revealed that expression of GRP78va is enhanced by ER stress and is notably elevated in human leukemic cells and leukemia patients. In contrast to the canonical GRP78 which is primarily an ER lumenal protein, GRP78va is devoid of the ER signaling peptide and is cytosolic. Through specific knockdown of endogenous GRP78va by siRNA without affecting canonical GRP78, we showed that GRP78va promotes cell survival under ER stress. We further demonstrated that GRP78va has the ability to regulate PERK signaling and that GRP78va is able to interact with and antagonize PERK inhibitor P58^IPK^. Our study describes the discovery of GRP78va, a novel cytosolic isoform of GRP78/BiP, and the first characterization of the modulation of UPR signaling via alternative splicing of nuclear pre-mRNA. Our study further reveals a novel survival mechanism in leukemic cells and other cell types where GRP78va is expressed.

## Introduction

Major advances have been made during the past decade in the understanding of how cells respond to impairment or saturation of the protein maturation machinery of the endoplasmic reticulum (ER). The unfolded protein response (UPR) signaling pathways are activated to reduce the unfolded protein load and at the same time increase protein folding capacity. The initiation of UPR is primarily regulated by the ER-transmembrane sensor proteins PERK, IRE1 and ATF6 [1], [2]. Upon ER stress, PERK activation leads to phosphorylation of the eukaryotic translation initiation factor eIF2α, which inhibits global protein biosynthesis while activating the translation of the transcription factor ATF4. Along with activated ATF6 and IRE1/XBP-1, ATF4 activates transcription of ER chaperones and folding enzymes, thus improving the protein processing capacity and alleviating protein aggregation in the ER.

A major ER chaperone is the 78 kDa glucose regulated protein GRP78, also referred to as BiP (immunoglobulin heavy chain binding protein) [3]–[5]. GRP78 belongs to the HSP70 protein family and is encoded by a single copy gene in both the human and mouse genome [6]. In contrast to the cytosolic counterparts of the HSP70 protein family, the amino-terminus of GRP78 contains a signal peptide that targets it to the ER and its carboxy-terminus contains the ER retention signal motif KDEL. The induction of GRP78 has been widely used as a sentinel marker for ER stress and UPR activation [7]. As an ER resident protein, GRP78 facilitates protein assembly, prevents intermediates from aggregation and targets misfolded proteins for degradation [8], [9]. In non-stressed mammalian cells, GRP78 binds to PERK, IRE1 and ATF6 and maintains them in an inactive form [10], [11]. GRP78 also interacts with pro-apoptotic molecules at the ER, blocking their activation [12]–[14]. When unfolded proteins pull GRP78 away from them, these pathways are activated, triggering the UPR, and if the stress is too severe, apoptosis. Therefore, as a key UPR regulator and target, GRP78 is postulated to play pivotal roles in pathological processes, however, the precise mechanisms remain to be determined [15]–[17].

Considering the central importance of UPR in regulating cell survival and death [1], [2], [18], [19], the prediction is that cells will evolve feedback regulatory pathways to modulate the key UPR executors. One such example is the ER stress induction of the DnaJ family protein P58^IPK^, first discovered as an inhibitor of the eIF2α protein kinase PKR in the cytosol [20]. P58^IPK^ serves as a negative feedback of UPR signaling by binding to the cytosolic domain of PERK and inhibiting its kinase activity, thereby terminating PERK activation in the late phase of the UPR [21]. As part of a protein complex with HSP70 at the cytosolic face of translocons, P58^IPK^ has been implicated as a mediator of co-translational ER protein degradation [22]. Nonetheless, P58^IPK^ was recently determined to be an ER lumenal protein, where it acts as a co-chaperone of GRP78 in protecting the stressed ER [23].

Alternative splicing is increasingly recognized as a crucial mechanism for generating protein isoforms with distinct cellular localization and sometimes antagonistic roles regulating survival and apoptotic processes, impacting various diseases including cancer [24], [25]. We discovered that the human and mouse *Grp78* allele contains conserved non-canonical splice sites and ER stress enhances the production of a variant form of GRP78 through retention of the first intron and internal translation initiation, referred to below as GRP78va. Among the various types of alternative pre-mRNA splicing, intron retention is relatively rare and few examples of biological consequence are documented [26]. Lacking the ER signal sequence, GRP78va is cytosolic and is notably elevated in human leukemic cell lines subjected to ER stress. The transcript of GRP78va is also present in AML, CML and ALL patients, but not in normal controls. GRP78va is a promoter of cell survival under ER stress. Through overexpression and siRNA knockdown studies, we further demonstrated that GRP78va has the ability to regulate PERK signaling and is able to interact with and act as an antagonist of PERK inhibitor P58^IPK^. Collectively, these studies provide the first evidence of the modulation of UPR signaling via alternative splicing of nuclear pre-mRNA and reveal a fundamentally new concept for a survival mechanism in leukemic cells and other cell types where GRP78va is expressed.

## Results

### Identification of a novel splicing variant of *Grp78* in mouse and human

**Figure 1:**
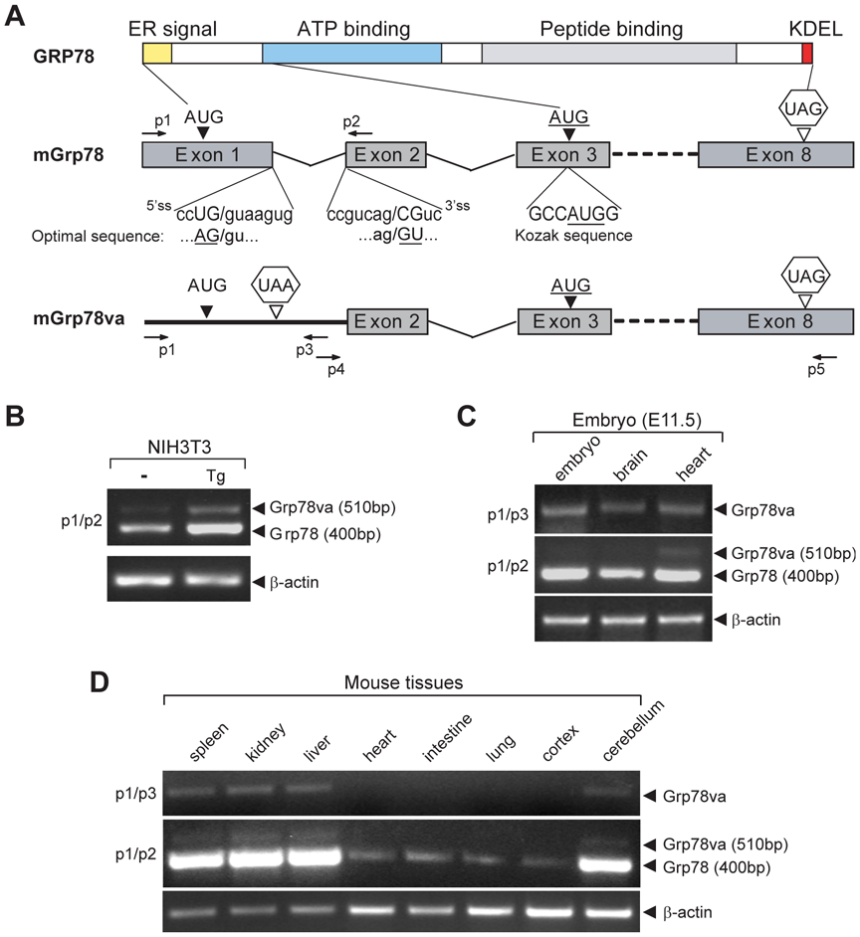
Detection of the alternative splicing transcript *Grp78va* of mouse *Grp78*. A. Schematic representation of GRP78 protein domains and canonical *mGrp78* and alternative transcripts. KDEL refers to the ER retention signal. Arrows labeled as p1 to p5 indicate positions of the primers used in RT-PCR. The sequence of 5′ splice site (5′ss) and 3′ splice site (3′ss) of intron 1 are aligned with the optimal splice site sequences. The translational initiation codon (AUG) and stop codon (UAG) of *Grp78* are located in exon 1 and exon 8 respectively. Intron 1 contains a stop codon UAA. The underlined AUG in exon 3 is a putative initiation site of *Grp78va* embedded in a consensus Kozak sequence. B. Detection of *Grp78va* transcript in mouse NIH3T3 cells by RT-PCR. NIH3T3 cells were either non-treated (−) or treated with 300 nM thapsigargin (Tg) for 16 hours and the total RNA were subjected to RT-PCR with the indicated primer sets. β-actin levels served as control. C. RT-PCR identification of *Grp78va* transcript in E11.5 mouse embryos and indicated organs. D. Detection of *Grp78va* transcript in adult mouse tissues. Total RNA extracted from the spleen, kidney, liver, heart, intestine, lung, cerebral cortex and cerebellum of a 5 wk old mouse were subjected to RT-PCR with the indicated primer sets.

GRP78 is a highly conserved ER chaperone protein. Critical domains include the ATPase catalytic domain and the peptide binding domain (Figure 1A). In both the mouse and human genome, GRP78 is encoded by a single-copy gene consisting of 8 exons. A novel splicing variant of *Grp78* (*Grp78va*) was discovered during RT-PCR of *Grp78* cDNA prepared from NIH3T3 cells, using primers spanning the 5′ end of exon 1 (p1) and the exon 1/2 junction (p2a) (Figure S1A). To our surprise, two PCR bands were detected, with the additional upper band clearly evident in cells treated with the ER stress inducer thapsigargin (Tg) (Figure S1B). Amplification of genomic DNA was ruled out in control PCR reactions performed with RNA as template (Figure S1B). Sequencing of the RT-PCR products revealed that the novel upper band contains an additional 112 bp intron 1 sequence which was spliced out in the canonical mouse *Grp78* transcript (Figure S1C). We determined that the fortuitous production of the novel PCR band could be due to the high sequence homology between the p2a primer complementary sequence at the exon 1/2 junction and the intron 1/exon 2 junction sequence (Figure S1D), which led to the amplification of both the canonical *Grp78* and the intron 1 retention variant form. The initial PCR results using primers p1/p2a were confirmed by using another set of primers (p1/p2), with p2 flanking the exon 2 of mouse *Grp78* (Figure 1A,B).

To further investigate the intron-retention variant form and determine whether it is expressed beyond cells in culture, two primer pairs (p1/p3; p4/p5) specific to the *Grp78va* transcript were designed, with both p3 and p4 located within intron 1 (Figure 1A). RT-PCR analysis showed that the *Grp78va* transcript was detected in E11.5 mouse embryos and embryonic brain and heart (Figure 1C). Interestingly, in adult tissues, the *Grp78va* transcript was readily detectable in mouse spleen, kidney, liver and cerebellum, and below detection limit in the heart, intestine, lung and cerebral cortex (Figure 1D). In every case, expression levels of *Grp78va* transcript directly parallel that of the canonical *Grp78*.

**Figure 2:**
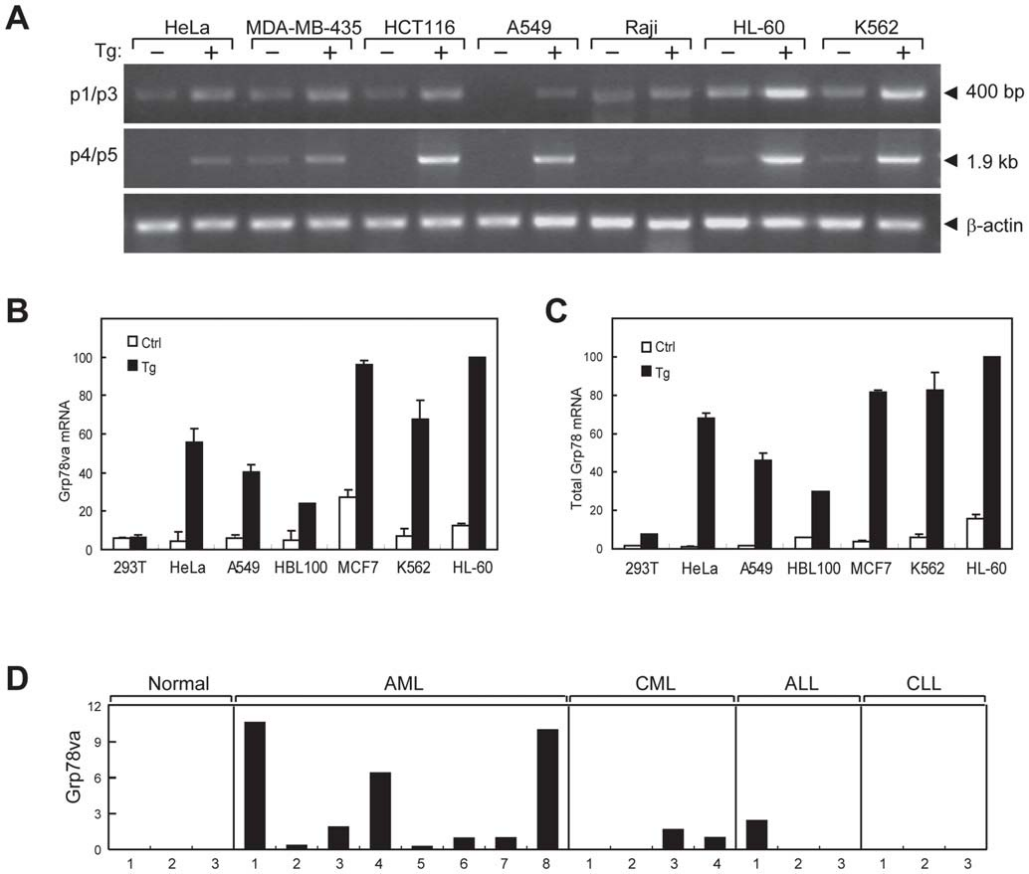
Identification of the *Grp78va* transcript in human cell lines and leukemia patient samples. A. Expression of the *Grp78va* transcript was determined by RT-PCR with the total RNA from the indicated human cell lines, which were either non-treated (−) or treated (+) with Tg (300 nM) for 16 hours. PCR was performed with specific primers for the human homologue of m*Grp78va* shown in Figure 1A. B,C. Quantitative real-time PCR to compare relative expression levels of *Grp78va* and total *Grp78* transcripts in different human cell lines respectively. D. Summary of *Grp78va* transcript expression in leukemia patients detected by RT-PCR. RNA samples from the indicated types of leukemia patients were used. β-actin levels served as control. The patient samples are either from peripheral blood or bone marrow (asterisk). The *Grp78* transcript levels after normalization against β-actin in normal individuals and leukemia patients are shown.

The *Grp78va* transcript was also detected in a variety of human monolayer and suspension cell lines, as determined by RT-PCR using primer pairs p1/p3 and p4/p5 of the human *Grp78* sequence (Figure 2A). As expected, Tg, which depletes the ER Ca^2+^ store, induced the canonical *Grp78* transcript, and the levels of *Grp78va* transcript were also increased in cells treated with Tg (Figure 2A,B). In addition, other ER stress inducers, the glycosylation inhibitor tunicamycin (Tun) and the proline acid analogue L-azetidine 2-carboxylic acid (AzC), known to induce canonical *Grp78* [3], also induced the expression of *Grp78va* (Figure S2). Utilizing quantitative real-time PCR, we further measured and compared the basal and Tg-induced levels of the *Grp78va* transcript (Figure 2B) and the total *Grp78* transcript levels in various human cell lines (Figure 2C). Notably, high basal level of *Grp78va* transcript was detected in the human breast cancer cell line MCF-7 and the human acute promyelocytic leukemia (APL) cell line HL-60. Among all the cell lines tested, HL-60 also expressed the highest level of total *Grp78* transcripts.

Sequencing of the PCR products shown in Figure 2A confirmed that the human *Grp78va* transcript contains the entire 97 bp intron 1 and eight intact exons of human *Grp78*. The splice donor and acceptor sites for intron 1 are conserved between mouse and human (Figure S1C), however, they do not match the optimal splice site sequences (Figure 1A). The general rules for intron retention are short intron size (around 100 bp) and suboptimal splice sites [27]. Interestingly, in addition to intron 1, intron 4 and 5 of the mouse and human *Grp78* also fit these criteria, however, RT-PCR analysis only identified retention for intron 1 and 4 (Table S1).

### Elevated *Grp78va* expression in ER-stressed leukemic cell lines and leukemia patients

Large-scale microarray using exon-exon junction probes has been used to monitor alternative splicing events in multi-exon human genes by comparing the junction probe intensity [28]. The microarray database deposited in NCBI/GEO contains the expression data of human *Grp78* from each exon-exon junction probe in 44 human tissue samples and 8 human cancer cell lines. Comparison of the intensities of exon 1/2 and exon 2/3 junction probes of *Grp78* in 52 samples included in this GEO profile revealed that the HL-60 and chronic myeloid leukemia (CML) K562 cell lines have the highest probability to express the intron 1 retention form of *Grp78*, while adult organs such as the heart, skeletal muscle and cerebral cortex are the most unlikely (Figure S3). The microarray analysis prediction that HL-60 and K562 cells express high levels of the *Grp78va* transcript whereas cerebral cortex and heart express low levels is in agreement with our measurements using real-time quantitative PCR (Figure 2B) and RT-PCR (Figure 1D) of selected human cell lines and mouse organs.

To further investigate the link between *Grp78va* and leukemia, patient bone marrow or peripheral blood samples were collected from patients diagnosed with AML, CML, ALL and CLL. The RT-PCR results determining the levels of *Grp78va* transcript are summarized in Figure 2D. The *Grp78va* transcript was expressed at various levels in AML, CML and ALL patients, but was below detection limit in normal controls and in CLL patients.

### The GRP78va isoform is a cytosolic protein

**Figure 3:**
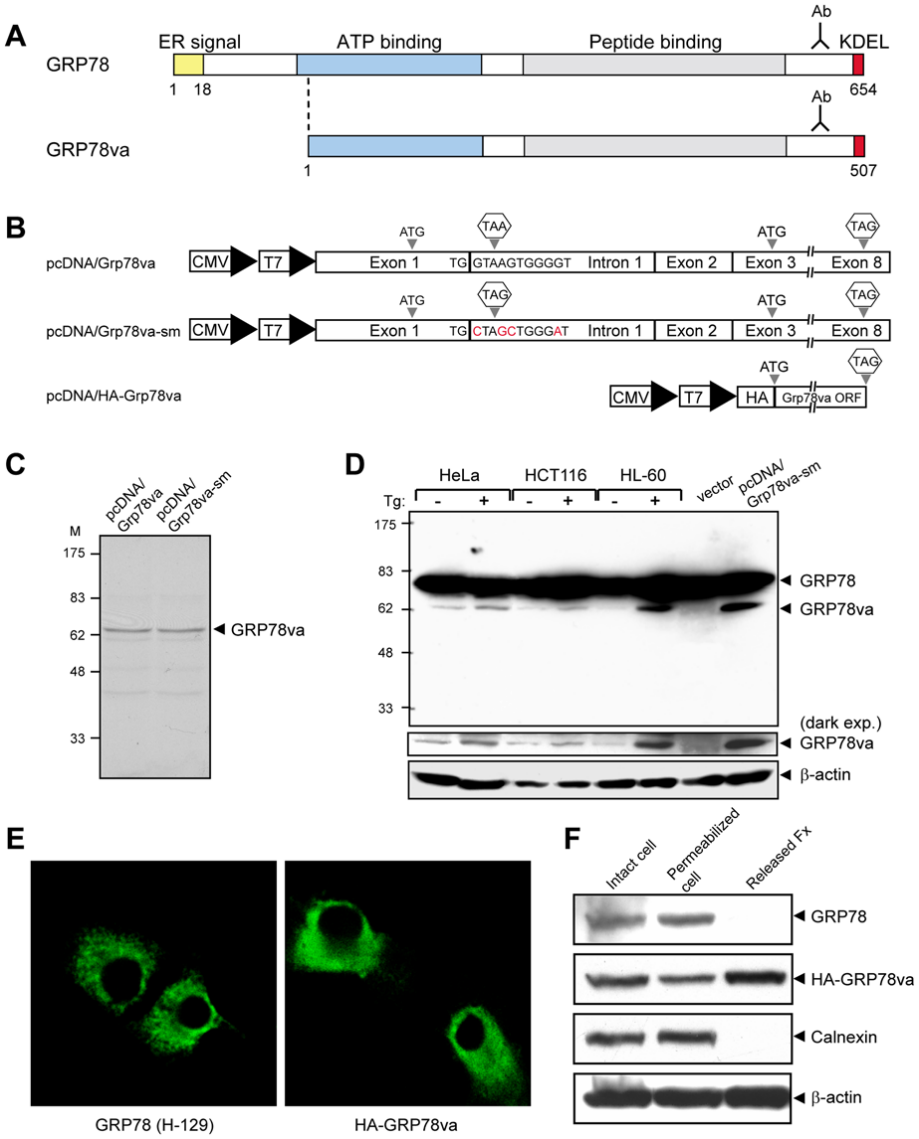
Characterization of human GRP78va protein. A. Schematic diagram comparing the protein domains of canonical GRP78 and GRP78va. The carboxy-terminal region recognized by the anti-GRP78 monoclonal antibody (Ab) is indicated. B. Schematic diagram of the indicated GRP78va expression plasmids. The base mutations in the 5′ splice site of intron 1 in pcDNA/Grp78va-sm are indicated in red. C. Coupled *in vitro* transcription and translation assay to detect the protein products from pcDNA/Grp78va and pcDNA/Grp78va-sm plasmids. D. Detection of endogenous GRP78va protein in human cell lines. The post-nuclear (PN) fractions from non-treated or Tg-treated HeLa, HCT116 and HL-60 cells were subjected to Western blot with the anti-GRP78 monoclonal antibody and β-actin served as loading control. GRP78va expression in 293T cells transfected with pcDNA/Grp78va-sm was used as positive control. A darker exposure of the GRP78va bands is shown below the entire blot. E. Immunofluorescence staining of GRP78 and GRP78va. HeLa cells were transfected with pcDNA-Grp78 (left panel) or pcDNA/HA-Grp78va (right panel). The cells were stained with anti-GRP78 antibody (H-129) or anti-HA antibody as indicated, followed by FITC-conjugated secondary antibodies. The confocal immunofluorescence images are shown. F. Cytosolic localization of GRP78va determined by the cell permeabilization assay. HeLa cells transiently transfected with pcDNA/HA-GRP78va were treated with 0.01% digitonin for 5 minutes. Intact cells, digitonin-permeabilized cells, and the released fraction were analyzed by Western blots for the proteins as indicated.

The translation of canonical GRP78 starts from the AUG initiation codon located in exon 1 (Figure 1A). However, retention of intron 1 introduces a UAA stop codon 123 bp downstream of the initial AUG. The next potential translation initiation site is an AUG containing sequence motif in exon 3, which matches the Kozak sequence (Figure 1A). Translation from this AUG site maintains the open reading frame of GRP78, generating a putative GRP78va protein of 507 a.a. (Figure 3A). Compared to GRP78, GRP78va is predicted to have a truncated amino-terminus and lack the ER-targeting signal, however, the remainder of GRP78va isoform will be identical to canonical GRP78 and recognizable by antibodies against the carboxy-terminus of GRP78. To test this, the human *Grp78va* cDNA was subcloned into the pcDNA3 expression plasmid driven by the CMV/T7 promoter (Figure 3B). This plasmid (pcDNA/Grp78va) was applied to a coupled *in vitro* transcription and translation assay to express GRP78va. A major protein band with a molecular weight of ∼62 kDa was detected (Figure 3C), which is consistent with the predicted molecular size of GRP78va. Additionally, to achieve higher expression level of GRP78va, pcDNA/Grp78va-sm was constructed where the 5′ splice donor site of intron 1 was mutated to inhibit residual splicing of intron 1 (Figure 3B). This mutated plasmid was able to express the 62 kDa GRP78va in the *in vitro* translation assay (Figure 3C), as well as in the transiently transfected 293T cells (Figure 3D, last lane).

To elucidate the subcellular localization of GRP78va, the expression plasmid pcDNA/HA-Grp78va was constructed to allow specific detection of the isoform protein with the HA-tag antibody (Figure 3B). Confocal immunofluorescence microscopy showed diffused cytosolic staining for HA-GRP78va, in contrast to the punctate perinuclear staining for GRP78 typical of ER staining (Figure 3E). Furthermore, digitonin release experiments were performed to differentiate the subcellular localization of canonical GRP78 and GRP78va, with calnexin, a transmembrane ER protein, serving as control for ER protein and β-actin serving as control for cytosolic protein. Our results showed that GRP78va and β-actin were released from the cell after disruption of the plasma membrane with low concentration (0.01%) of digitonin, but not canonical GRP78 or calnexin (Figure 3F), confirming that GRP78va is a cytosolic protein.

To monitor endogenous GRP78va expression in different human cell lines, post-nuclear (PN) cell lysates were prepared from HeLa, HCT116 and HL-60 cells to enrich for the cytosolic isoform GRP78va. Western blot analyses were performed with the PN fractions from Tg-treated or non-treated cells, with ectopically expressed GRP78va in 293T cells indicating the size of the isoform protein (Figure 3D). In all three cell lines, basal GRP78va expression was detectable, and corresponding with the increase of *Grp78va* transcript after Tg stress (Figure 2B), GRP78va protein level was also increased in the Tg-treated cells (Figure 3D). Consistent with GRP78va being truncated at the amino-terminus, antibody against the amino-terminus of GRP78 was unable to detect GRP78va (Figure S4). Further, to rule out the possibility that GRP78va is a degraded form of GRP78, a siRNA targeting a 21-nucleotide sequence unique to the retained intron 1 of human *Grp78va* was utilized to specifically knock down the *Grp78va* transcript (Figure S5A). In cells transfected with siGrp78va, the level of canonical GRP78 was unchanged at both mRNA and protein levels whereas the level of GRP78va was substantially reduced at both mRNA and protein levels (Figure S5B-D). This, coupled with the different cellular localization for GRP78va and GRP78, indicates that GRP78va is a product of the *Grp78va* transcript and not a degraded form of GRP78.

### GRP78va specifically enhances PERK signaling in the unfolded protein response

**Figure 4:**
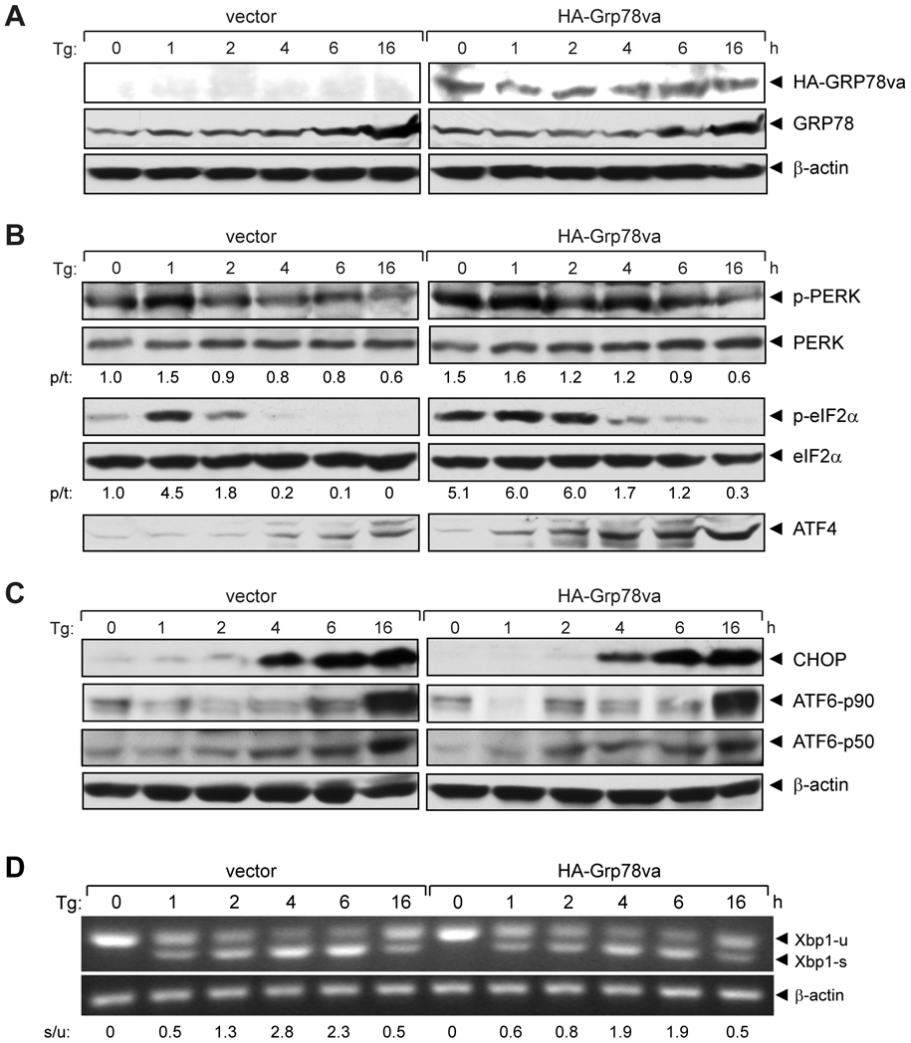
GRP78va enhances PERK signaling. A. Western blots to confirm the ectopical expression of GRP78va. HeLa cells stably transfected with vector or pcDNA/HA-Grp78va were treated with Tg (300 nM) for the indicated time. HA-GRP78va and endogenous GRP78 were detected by anti-HA and anti-GRP78 antibody respectively. β-actin levels served as loading control. B. Western blots were performed to detect the kinetics and magnitude of UPR signaling markers identified on the right following Tg treatment for the indicated time points. The experiments were repeated twice. The representative results are shown with the ratio (p/t) of p-PERK/PERK or p-eIF2α/eIF2α indicated under each panel with the non-treated control cells set as 1.0. C. Detection of the CHOP activation and ATF6 cleavage following Tg treatment for the indicated time points. The uncleaved (p90) and cleaved (p50) forms of ATF6 are indicated. β-actin levels served as loading control. D. RT-PCR to detect *Xbp1* splicing following Tg treatment for the indicated time. The positions of the *Xbp1* unspliced (u) and spliced (s) forms were indicated and the ratio of spliced to unspliced form (s/u) is indicated below. The experiments were repeated three times and the representative results are shown.

Canonical GRP78 is known to interact and regulate the activation of the three major UPR pathways: PERK, IRE1 and ATF6. As a first step to determine the biological function of GRP78va, HeLa cell lines stably transfected with pcDNA/HA-Grp78va or the empty vector were established. Western blot results showed that stable overexpression of GRP78va did not significantly affect the basal level or Tg-induction of canonical GRP78 (Figure 4A). The same cell lysates were analyzed for the status of the UPR pathways. As shown in Figure 4B, overexpression of GRP78va strongly activated the ER eIF2α kinase, PERK, as evidenced by increased phosphorylation of PERK, even in the absence of ER stress (time 0). Following Tg treatment, PERK activation was sustained longer than in control cells transfected with the empty vector. This correlates with enhanced and sustained eIF2α phosphorylation and higher level of ATF4 upon Tg treatment (Figure 4B). In contrast, we did not observe any significant change in ATF6 activation, as measured by proteolytic cleavage, which may explain why Tg induction of CHOP, co-regulated by ATF6 and ATF4, was not enhanced in the GRP78va overexpressed cells (Figure 4C). ER stress activates the kinase and ribonuclease activities of IRE1, which removes a 26-nucleotide intron from *Xbp1* mRNA (Xbp1-u), giving rise to a spliced mRNA (Xbp1-s). While the kinetics of *Xbp1* splicing was similar in GRP78va overexpressed and control cells, the ratio of spliced versus unspliced *Xbp1* was slightly lower at 2 to 6 hours in GRP78va overexpressed cells than in control cells (Figure 4D).

**Figure 5:**
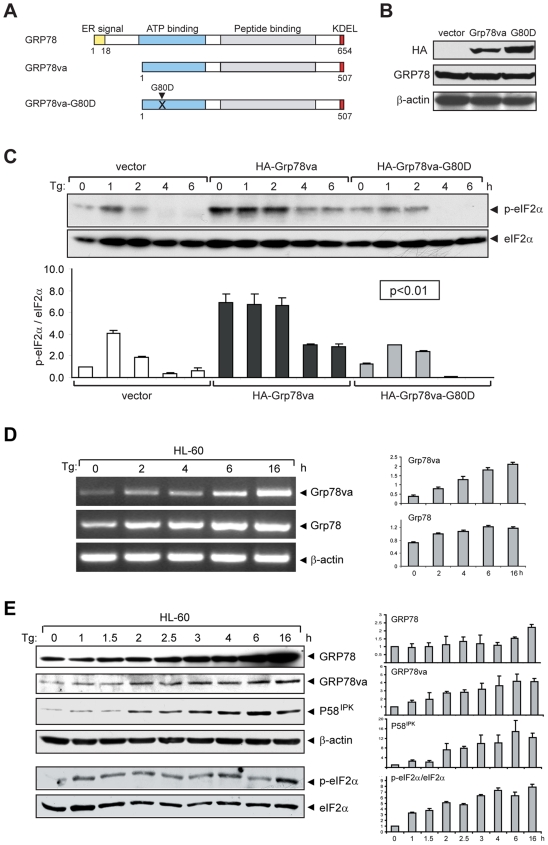
Mutation in ATP binding domain of GRP78va attenuates eIF2α phosphorylation. A. Schematic representation of human GRP78, GRP78va, and the ATP binding mutant GRP78va-G80D. The position of mutation site is indicated. B. Western blots to detect ectopic expression of GRP78va and the mutant in stably transfected HeLa cell lines. The GRP78va proteins and endogenous canonical GRP78 were detected by the anti-HA and anti-GRP78 antibody respectively, with β-actin as loading control. C. Time course analysis of eIF2α phosphorylation in Tg-treated HeLa cells stably expressing HA-GRP78va or the mutant. Western blots were performed to determine total and phospho-eIF2α levels (upper panel). The ratio of phospho-eIF2α to total eIF2α from two independent experiments are summarized and expressed as the mean with the indicated standard deviation (SD) (lower panel). P values for all time points are indicated. D. Time course analyses of Tg induction of *Grp78va* and canonical *Grp78* transcripts by RT-PCR. HL-60 cells were treated with Tg (300 nM) for the indicated time and the total RNA was subjected to RT-PCR (left panel). The experiments were repeated three times. The results are summarized and expressed as the mean of the normalized *Grp78va* or canonical *Grp78* levels with the indicated SD (right panel). E. Time course analyses of Tg induction of canonical GRP78, GRP78va, P58^IPK^ and eIF2α phosphorylation (p-eIF2α). HL-60 cells treated with Tg (300 nM) for the indicated time were used for Western blot for detection of the indicated proteins with β-actin as loading control (left panel). The experiments were repeated twice. The levels of canonical GRP78, GRP78va and P58^IPK^ normalized to β-actin and the ratio of phospho-eIF2α to total eIF2α are summarized and plotted respectively (right panel).

The ATP binding domain of GRP78 is critical for its catalytic activity, and a single amino acid substitution G227D produced an ATP binding mutant [29]. Stable HeLa cell lines were established with this corresponding mutation in GRP78va (G80D) and the expression level of the mutant form was assayed in parallel with canonical GRP78va by Western blot (Figure 5A,B). Despite a slightly higher expression level of the mutant, it did not lead to enhanced eIF2α phosphorylation in non-stressed (time 0) or Tg-treated cells as was observed in the GRP78va overexpressed cells (Figure 5C). These results establish that the ATP binding activity of GRP78va is essential for its positive regulatory function and further excludes the possibility that GRP78va overexpression fortuitously triggered PERK signaling as a consequence of ectopic protein expression.

To investigate further the relationship between endogenous GRP78va and PERK activation, we first monitored in HL-60 cells the kinetics of Tg-induced expression of GRP78va and phosho-eIF2α as a monitor for PERK activation. GRP78 and total eIF2α levels were also measured in parallel. Representative gels/blots and a summary of quantified results from multiple experiments are shown. The upregulation of GRP78va started at an early stage (∼2 hours) following Tg treatment, at both the mRNA (Figure 5D) and protein levels (Figure 5E) and the increase was sustained for 16 hours. Interestingly, P58^IPK^, a known inhibitor of PERK, was also quickly induced (from 2 hours) during the same treatment (Figure 5E), which is different from the previous observation that P58^IPK^ is induced at late phase (12 hours) of tunicamycin-induced ER stress in NIH3T3 cells [30]. Elevation of eIF2α phosphorylation was evident within the first hour and sustained throughout the treatment period (Figure 5E).

### GRP78va interacts with P58^IPK^ and reduces its protein level

**Figure 6:**
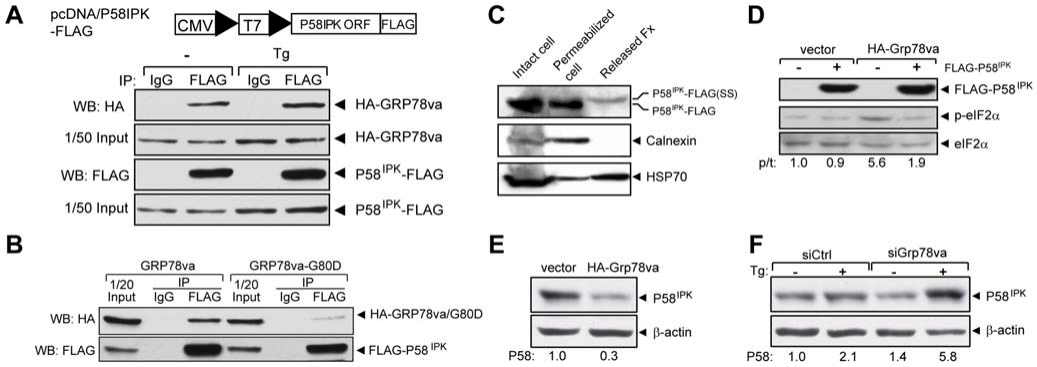
Physical and functional interactions between GRP78va and P58^IPK^. A. Co-immunoprecipitation of GRP78va with P58^IPK^. 293T cells co-transfected with pcDNA/P58^IPK^-FLAG (schematic drawing shown above) and pcDNA/HA-Grp78va were either non-treated (−) or treated with Tg (300 nM) for 16 hours. Immunoprecipitations were performed with either anti-FLAG antibody or control mouse IgG, followed by Western blot with the respective antibodies. The input levels are shown. B. Reduced binding between FLAG-P58^IPK^ and ATP binding mutant of GRP78va. 293T cells were co-transfected with pcDNA/FLAG-P58^IPK^ and pcDNA/HA-Grp78va or the mutant Grp78va-G80D. Immunoprecipitations were performed with anti-FLAG antibody or control IgG, followed by Western blot. C. Cytosolic localization of P58^IPK^ determined by the cell permeabilization assay. HeLa cells transiently transfected with pcDNA/P58^IPK^-FLAG were permeabilized by 0.01% digitonin for 5 minutes. The various fractions were subjected to Western blots for the proteins as indicated. P58^IPK^-FLAG(SS) designates P58^IPK^ with a slower electrophoretic mobility being released from the permeabilized cells, consistent with retention of signal sequence. D. Overexpression of FLAG-P58^IPK^ suppressed eIF2α phosphorylation mediated by GRP78va. Following transient transfection of the P58^IPK^ expression plasmid or vector into the indicated stably transfected HeLa cell lines, Western blots were performed to probe for the indicated proteins. The experiments were repeated twice. The representative Western blots are shown with the ratio (p/t) of phospho-eIF2α to total eIF2α indicated. E. Overexpression of GRP78va reduced endogenous P58^IPK^ levels. Western blots were performed in HeLa cells stably transfected with vector or HA-GRP78va expression plasmid. The experiments were repeated three times. The representative Western blots are shown and the P58^IPK^ levels normalized to β-actin are indicated below. F. Knockdown of GRP78va increased endogenous P58^IPK^ level. Western blots were performed with HeLa cells transfected with control siRNA (siCtrl) or *Grp78va* siRNA (siGrp78va) to detect endogenous P58^IPK^ and β-actin level. The cells were either non-treated (−) or treated (+) with Tg (300 nM) for 16 hours. The P58^IPK^ levels normalized to β-actin are shown below the immunoblots. Statistical comparisons were made between Tg-treated cells transfected with siCtrl or siGrp78va, p = 0.03.

P58^IPK^ is an inhibitor of PERK acting through the cytosolic kinase domain of PERK and knockdown of P58^IPK^ constitutively activates PERK [21]. Since GRP78va activates PERK signaling, we tested whether GRP78va diminishes the P58^IPK^ inhibitory effect on PERK through protein-protein interaction. Co-immunoprecipitation was performed with lysates from 293T cells co-transfected with HA-GRP78va and P58^IPK^-FLAG, where the FLAG-tag was fused to the ORF of P58^IPK^ at the carboxy-terminus, so that it does not interfere with the amino-terminal ER signal peptide (Figure 6A). GRP78va was co-immunoprecipitated with P58^IPK^ in both non-stressed and Tg-treated cells. Co-immunoprecipitation of GRP78va was also observed with FLAG-P58^IPK^ where the FLAG-tag was fused to the amino-terminus of P58^IPK^ (Figure 6B). Furthermore, the interaction between P58^IPK^ and GRP78va requires the ATP binding function of GRP78va as the binding of the ATP binding mutant GRP78va-G80D to P58^IPK^ was much weaker compared to wild-type GRP78va (Figure 6B).

Since GRP78va is a cytosolic protein, its interaction with P58^IPK^ is expected to occur in the cytosolic compartment. While originally discovered as a cytosolic protein [20], [30], recent evidence suggests that most P58^IPK^ is located in the ER lumen as a co-chaperone of ER lumenal GRP78 [23], [31], however its weak signal sequence allows for inefficient translocation and may therefore generate some cytosolic forms [23]. Through confocal microscopy using PDI staining as an ER marker, we observed that while P58^IPK^-FLAG co-localized with PDI in the ER, it was also detected in the cytosol (Figure S6). The existence of a cytosolic pool of P58^IPK^ was further confirmed by digitonin release experiment, with the heat shock protein HSP70 serving as control for cytosolic protein and calnexin serving as control for ER protein. In HeLa cells transfected with P58^IPK^-FLAG, we observed that P58^IPK^ and HSP70, but not calnexin, was detected in the released fraction when the cells were treated with low concentration (0.01%) of digitonin (Figure 6C). Additionally, the electrophoretic mobility of the released form of P58^IPK^ was slower than the form that was retained inside the permeabilized cells, consistent with previous reports that P58^IPK^ bearing the signal peptide migrated slower than the ER form in SDS gel [23], [31] and the weak P58^IPK^ signal sequence allows for inefficient translocation into the ER [23]. Thus, both imaging and biochemical studies suggest that a subfraction of P58^IPK^ can exist in the cytosolic compartment.

Next we examined whether there is functional interaction between GRP78va and P58^IPK^. In HeLa cells stably expressing GRP78va, the level of phospho-eIF2α level was increased by 5.6-fold compared to vector control; however, upon co-expression with P58^IPK^, this level was reduced to 1.9-fold; whereas overexpression of P58^IPK^ alone had no effect on phospho-eIF2α level (Figure 6D). This implies that P58^IPK^ is an antagonist of GRP78va. Conversely, GRP78va is an antagonist of P58^IPK^ since stable overexpression of GRP78va in HeLa cells decreased endogenous P58^IPK^ by 70% (Figure 6E). Further, modulation of endogenous P58^IPK^ by endogenous GRP78va in Tg treated cells was demonstrated by a 2.8-fold increase in P58^IPK^ level where GRP78va was down-regulated by siRNA (Figure 6F).

### GRP78va protects leukemic cells against ER stress-induced cell death

**Figure 7:**
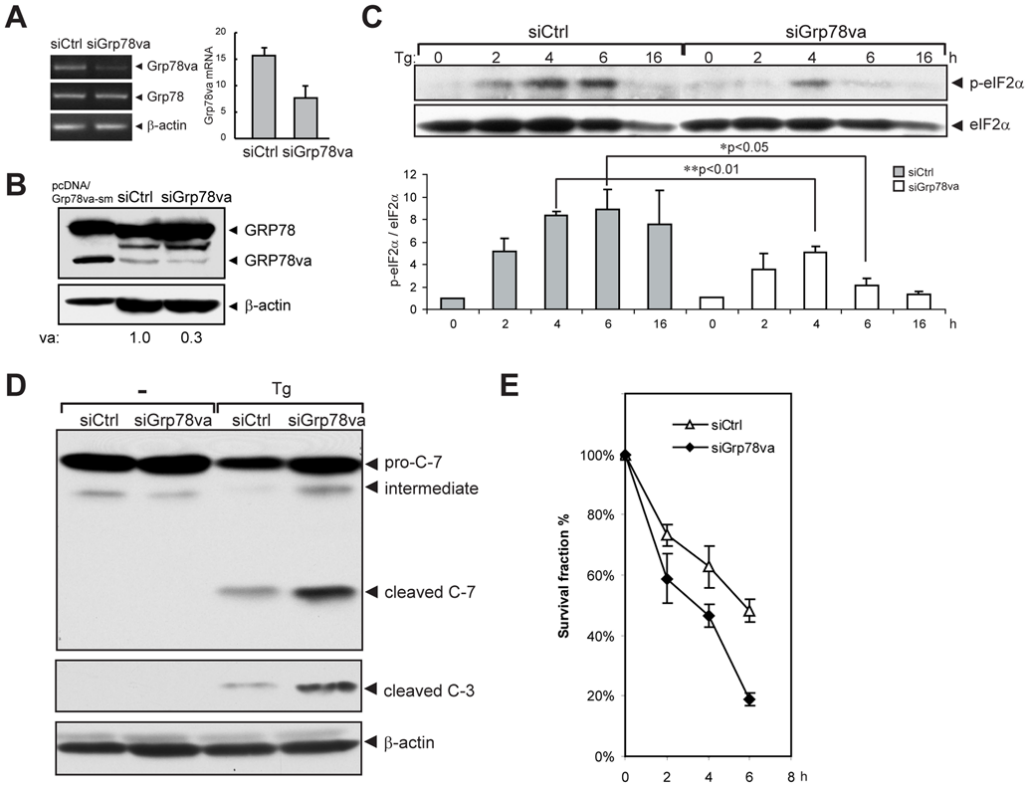
GRP78va protects HL-60 cells from ER stress-induced cell death. A, B. Knockdown of GRP78va in HL-60 cells. RT-PCR (A, left panel) or quantitative real-time PCR (A, right panel) was used to determine the level of *Grp78va* transcript in HL-60 cells transfected with the indicated siRNA. Western blot. B was performed to determine the levels of the proteins indicated. C. Inhibition of Tg-induced eIF2α phosphorylation by knockdown of GRP78va. HL-60 cells were transfected with either siCtrl or siGrp78va. After treatment with Tg (300 nM) for the indicated time, phospho-eIF2α and total eIF2α were detected by Western blot (upper panel). The experiments were repeated three times. The results are summarized and the ratio of p-eIF2α/eIF2α is plotted with SD (lower panel). The ratio of the non-treated, siCtrl transfected cells was set as 1. P values are indicated. D. Western blot of caspase-7, cleaved caspase-3 and β-actin in siRNA-transfected HL-60 cells treated with Tg for 24 hours. E. Soft-agar clonogenic survival assay for HL-60 cells after transfection with the indicated siRNA for 72 hours and then treated with Tg (300 nM) for the indicated time.

Translational attenuation mediated by eIF2α phosphorylation is a major pro-survival mechanism for the UPR and promotes tumor cell adaptation to stress [32], [33]. Given that leukemia is a highly malignant disease, GRP78va expression in leukemic cells may regulate PERK signaling and offer protection against ER stress-induced apoptosis, leading to survival. To test this, HL-60 cells were transfected with siGrp78va or control siRNA, and specific knockdown of GRP78va at the transcript (Figure 7A) and protein level (Figure 7B) was confirmed. Compared to control siRNA, cells transfected with siGrp78va showed significant attenuation of eIF2α phosphorylation induced by Tg treatment (Figure 7C). Western blot analyses further showed increased levels of the cleaved products of caspase-3 and -7 in Tg-treated cells transfected with siGrp78va, as compared to control siRNA (Figure 7D), corresponding with decrease in cell survival as measured by soft agar clonogenic assay in cells where GRP78va was knocked down (Figure 7E). Similarly, in colony survival assays, knockdown of GRP78va reduced viability of Tg-treated HeLa cells (Figure S7A), whereas overexpression of GRP78va enhanced cell survival (Figure S7B).

## Discussion

Alternative splicing is a major mechanism for generating protein diversity, expressing proteins that are related but with diverse function and localization. Interestingly, a large number of apoptotic regulators undergo alternative splicing, suggesting that this is a crucial mechanism for survival and death decisions [24]. GRP78/BiP, which represents a major anti-apoptotic arm of the UPR, has been traditionally regarded as an ER lumen chaperone protein [4], [16]. Recently, GRP78 has also been localized on the cell surface [34], [35]. Although the amount of surface GRP78 represents only a very small fraction of cellular GRP78, it has been implicated in enhancing mitogenic signaling, proliferation and cell survival [15], [17], [36]–[40]. Here, we report the discovery of GRP78va, a novel cytosolic isoform of GRP78 generated by alternative splicing of *Grp78* nuclear pre-mRNA. Our findings suggest the ability of GRP78va to mediate a previously unanticipated positive feedback regulation of PERK, potentially via cytosolic interaction with P58^IPK^. Our studies reveal several novel observations which may have important clinical implications in leukemia and other pathophysiological conditions that express this isoform.

Why has GRP78va escaped detection despite extensive studies on the structure and function of GRP78/BiP over the past 25 years? Difficulty to detect the isoform proteins generated from alternative spliced mRNAs could be due to transcript instability, reduced translation efficiency or instability of the new proteins. The abundance of *Grp78va* mRNA may be affected by nonsense-mediated RNA decay (NMD). NMD is a quality-control mechanism that selectively degrades mRNAs harboring premature termination (nonsense) codons. Most recently, it was reported that NMD is inhibited in ER-stressed cells and this inhibition is dependent on eIF2α phosphorylation [41]. The repression of NMD stabilizes the target mRNAs, including the UPR factors ATF4, ATF3 and CHOP, and thereby arguments the cellular stress response. With the introduction of a premature stop codon by intron 1 retention, *Grp78va* mRNA is a potential NMD target. Upon ER stress, inhibition of NMD could lead to stabilization of *Grp78va* mRNA and translation of functional GRP78va protein. Since GRP78va initiates by an internal AUG that is also contained in the canonical GRP78 transcript, the issue arises whether internal initiation might also occur. In our experimental design, the siRNA against GRP78va specifically targets the retained intron 1 of the *Grp78va* transcript, thereby affecting only translation from the *Grp78va* transcript but not internal initiation from the fully spliced, canonical *Grp78* transcript. We observed that the decrease in GRP78va protein level generally corresponds with the decrease in the *Grp78va* transcript level in the siGrp78va-treated cells (Figure S5), suggesting that the majority of GRP78va results from *Grp78va* mRNA translation. At the post-translational level, HA-GRP78va, was stabilized by the proteasome inhibitor MG115, whereas endogenous GRP78 was not affected (Figure S8A), consistent with a much shorter half life for GRP78va compared to canonical GRP78, as determined by the cycloheximide chase experiment (Figure S8B). Analysis of the protein amino acid sequence revealed a potential PEST-rich region at the carboxy-terminus of GRP78va (Figure S8C). PEST sequences are present in regulatory proteins, such as transcription factors, protein kinases or phosphatases and cyclins, and often locate at carboxy-terminus in these proteins, serving as a proteolytic signal contributing to rapid degradation of proteins [42], [43]. While canonical GRP78 also contains the same PEST sequence, the ER compartment where it is localized likely protects it from recognition and degradation by ubiquitin-proteasome system. Here we report that with the proper PCR primers, the alternatively spliced *Grp78va* transcript is readily detectable in mouse embryos, specific adult tissues, as well as peripheral blood and bone marrow samples from leukemic patients. The GRP78va expression pattern matches well with predictions from the genome-wide exon junction microarray [28] and is represented, albeit rarely, in the current expressed sequence tag (EST) database which tends to bias against less abundant alternative forms. While GRP78va may have diverse cellular function which awaits future investigation, here we establish that GRP78va exhibits anti-apoptotic activity against ER stress.

How might GRP78va protect cells from ER stress induced cell death? In contrast to canonical GRP78, GRP78va is cytosolic due to the lack of the amino-terminal ER signal sequence. Based on previous studies of GRP78 mutants, the GRP78va isoform corresponds to the protein initiating at Met-148/153 which showed greater ATP binding than the wild-type GRP78 [44]. Thus, the conservation of both the ATP binding and the peptide binding domains in GRP78va suggests that it can act as a cytosolic counterpart of canonical GRP78, interacting with client proteins playing critical roles in cell survival. One key function of the ER localized canonical GRP78 is its ability to regulate ER stress signaling by maintaining ER transmembrane sensors PERK, IRE1 and ATF6 in their inactive state in non-stressed cells. As a first test of GRP78va function, we examined whether it affects ER stress signaling pathways. Using both overexpression and knockdown approaches, we discovered that GRP78va selectively upregulates PERK signaling. Further, point mutation of GRP78va defective in ATP binding greatly diminished its regulatory effect on induction of eIF2α phosphorylation, suggesting that GRP78va acts through its interaction with cytosolic client protein(s) capable of modulating PERK activity.

Here we identified P58^IPK^ as the first client protein of GRP78va. There are some discrepancies in literature regarding the subcellular localization of P58^IPK^, as its activity has been documented in both the cytosolic and the ER compartments. P58^IPK^ was originally discovered as a cytosolic protein but later discovered to possess a weak ER signal peptide sequence [20], [23], [30]. There are precedents in literature where an ER protein can also localize in the cytosol. Potential mechanisms include inefficient translocation due to weak signal peptide sequence [45], retrotranslocation from the ER lumen to the cytosol after peptide cleavage inside the ER [46]; after signal peptide cleavage but prior to ER insertion, translocation can be aborted and the product released into the cytosol [47]; and retrotranslocation from the ER lumen to the cytosol via ERAD but escapes proteasome degradation [48]. Further, different cell lines exhibit wide variations in the extent of relocation [46]. Results from our studies in HeLa cells are consistent with insufficient translocation of P58^IPK^ due to weak signal sequence [23], and provide a plausible explanation for the original role of P58^IPK^ as an inhibitor of the cytosolic kinase activities of PERK and PKR [20], [30], as a cytosolic component of the translocon [22], and the recent finding that P58^IPK^ regulates influenza virus mRNA translation and replication through a PKR-mediated mechanism in the cytosol [49]. Further, as the minor cytosolic population of P58^IPK^ is able to inhibit the low abundance PERK or PKR kinase, this inhibition could be suppressed by low amount GRP78va through antagonizing the cytosolic P58^IPK^. In addition, the distribution of P58^IPK^ between the ER and cytosol could be different in different cell types, such as mouse embryonic fibroblasts (MEFs) versus human cancer cells, or under different cellular conditions, such as normal physiological versus pathological conditions. Future investigations will be required to resolve how these factors contribute to P58^IPK^ subcellular localization and function.

Our results also predict that other cytosolic co-chaperone proteins might associate with GRP78va and assist its function. It is known that Hsp70 proteins employ DnaJ family proteins as co-chaperones, which preferentially bind to the ATP form of their partner Hsp70 and stimulate the ATPase activity to stabilize Hsp70-polypeptide complexes [50], [51]. DnaJ/Hsp40 chaperone proteins interact with the conserved ATPase domain of HSP70 proteins through their conserved J-domain. As the ER lumenal HSP70 chaperone, GRP78 is known to interact with a group of DnaJ/Hsp40 proteins in the ER lumen, such as ERdjs [52]–[54]. Since GRP78va retains most part of ATPase domain of canonical GRP78, it is expected to potentially bind other cytosolic chaperones, such as DnaJ/Hsp40 proteins besides P58^IPK^. Further experiments are necessary to characterize the cytosolic co-chaperone proteins of GRP78va. It is also tempting to speculate that GRP78va might act as a cytosolic chaperone collaborating with its cytosolic co-chaperones to promote the folding of nascent cytosolic polypeptides, since it corresponds to the GRP78 mutant initiated from Met-148 in previous study and exhibits strong ATP binding activity [44], implicating that GRP78va has the ability to bind to client proteins.

Induction of GRP78 and activation of PERK signaling represents two major pro-survival pathways in the UPR. GRP78 promotes ER protein folding and efficient degradation of aggregated proteins through ERAD, which alleviates the unfolded protein load in the ER and increases cell survival. Likewise, PERK-mediated eIF2α phosphorylation is protective by attenuating the protein biosynthesis and consequently reducing the total protein load on the ER. Thus, both GRP78 upregulation and PERK activation promote cell survival and adaptation to pathophysiological stress [5], [15], [32], [33], [55], [56]. P58^IPK^ is inducible by ER stress. The cells employ the cytosolic population of P58^IPK^ to inhibit PERK activity, allowing the cells to recover from global translational inhibition. Our data suggests that ER stress also induces GRP78va as a cytosolic counterpart of GRP78, and a potential feedback mechanism to regulate P58^IPK^. Through complex formation with cytosolic P58^IPK^, GRP78va may act as an antagonist of P58^IPK^. Thus, when GRP78va is overexpressed, the level of P58^IPK^ was decreased and PERK signaling was enhanced. Therefore, GRP78va has the ability to function as a novel regulator of PERK signaling under ER stress and this regulation may play an important role especially in cancer cells where GRP78va is highly expressed. However, since sustained PERK activation could be harmful [57] and persistent ER stress attenuates survival pathways mediated by IRE1 and ATF6 signaling [19], this may explain why GRP78va expression might be stringently regulated at the transcript stability level, as well as at the post-translational level.

Aberrant splicing has been found to be associated with various diseases, including cancer [25]. In this study, through microarray and biochemical analysis, we discovered that leukemic cell lines and leukemia patients expressed high levels of GRP78va. Evidence suggests that alteration of phosphorylation and/or expression of proteins that recognize splice sites and assembly of the splicesome in leukemia can be caused by aberrant BCR/ABL fusion protein, deregulated expression of DNA topoisomerase I or the hematopoietic transcription factor Spi-1/PU.1 [58], [59]. Thus, a higher incidence of intron retention of *Grp78* in leukemic patients may be due to alteration of the pre-mRNA splicing machinery. On the other hand, genomic mutations associated with oncogenesis may also interfere with the recognition and selection of splice sites and affect pre-mRNA splicing pattern.

Considering the high expression of GRP78va in leukemia and its pro-survival role against ER stress, how might leukemic cells experience ER stress? Recent reports suggest that retroviral infection, the pathologically expressed fusion protein PML-RARα and reactive oxygen species can induce ER stress in leukemic cells [60], [61]. Therapeutic drugs in clinical treatment or pre-clinical test, such as arsenic trioxide and imatinib mesylate, have been shown to upregulate ER stress markers and induce leukemic cell death through ER stress-mediated apoptosis [62]. Further, proteomic analysis discovered that GRP78 is differentially expressed in the hematopoietic stem cell (HSC)-like fractions from the bone marrow of leukemia patients [63]. It will be interesting to determine if GRP78va is also elevated in parallel with GRP78 in the HSC cell fractions. While GRP78 is established to be critical for solid tumor progression and drug resistance [5], [15], [55], [64]–[69], the role of GRP78 and GRP78va in hematological cancers remains to be determined. The discovery of the novel cytosolic isoform GRP78va points to a new paradigm for how GRP78 functions in regulating cell homeostasis and future identification of other client proteins of GRP78va will yield new insight on how GRP78 and UPR signaling regulate cell survival and death beyond the ER compartment.

## Materials and Methods

### Cell culture

Human cell lines HeLa, MDA-MB-435, HCT116, A549, MCF-7, HBL100, 293T and mouse fibroblast NIH3T3 cells were cultured in Dulbecco's modified Eagle medium (DMEM) containing 10% fetal bovine serum and 1% penicillin/streptomycin. Human lymphoma cell line Raji, APL cell line HL-60 and CML cell line K562 were maintained in RPMI 1640 medium supplemented with 10% fetal bovine serum and 1% penicillin/streptomycin.

### RT-PCR and quantitative real-time PCR

Total RNA from cell cultures, mouse embryos and tissues was extracted by Trizol reagent (Invitrogen) and treated with DNase I before converted to cDNA by using SuperScript II reverse transcriptase (Invitrogen) with oligo d(T) primer following the manufacturer's instruction. For quantitative real-time PCR, cDNA samples from human cell lines were analyzed in triplicate with the SYBR® Green Supermix (BioRad) using iCycler Real-Time PCR Detection System (BioRad). All primer sets were tested for PCR efficiency as recommended by the manufacturer's instruction. A standard curve was prepared for each set of primers using serial titration of the cDNA samples. The relative mRNA level was calculated from primer-specific standard curves using the iCycler Data Analysis Software and normalized to GAPDH levels. Specific PCR primers for *Grp78* or *Grp78va* are indicated in Figure 1A and the sequence of primers are listed in the Supporting Information S1.

### cDNA cloning and plasmid construction

Bridging-based two round PCR was used to amplify human *Grp78va* cDNA sequence and the cloning procedure for the construction of all the expression plasmids were described in the Supporting Information S1. All the clones were verified by sequencing.

### Clinical specimens

Leukemic blasts from bone marrow and peripheral blood samples were generously provided by Dr. Allen Yang (University of Southern California Keck School of Medicine). They were obtained from diagnosed leukemia patients in USC/Norris Comprehensive Cancer Center according to institutional guidelines. The ethical use of the human tissues for research was approved by the University of Southern California Institutional Review Board. Written consent was obtained.

### *In vitro* transcription and translation assay

One µg of the template plasmid DNA was applied in the TNT® quick coupled transcription/translation assay (Promega) following the manufacturer's protocol.

### Transfection methods

For transfection of plasmids, Polyfect reagent (Qiagen) was used following the manufacturer's protocol. The stable cell lines were established by transfecting the expression plasmids into HeLa cells, followed by selection with G418 (400 µg/ml, Gibco) for 2 weeks. Individual clones were picked, trypsinized and seeded to 24 well-plate for further culture in fresh DMEM medium supplemented with G418 (200 µg/ml). For transfection of siRNA, Lipofectamine™ 2000 reagent (Invitrogen) was used following manufacturer's protocol. The sequence of siRNA against *Grp78va* and control siRNA were described in the Supporting Information S1.

### Preparation of post-nuclear lysate

Cells were resuspended in ice cold NE buffer A (10 mM Hepes, pH 7.9, 10 mM KCl, 1.5 mM MgCl_2_, 10 mM EDTA, 0.35% NP-40, 1x protease inhibitor complex), rotated at 4°C for 10 minutes and centrifuged to collect the supernatant as the PN fraction, which were stored in −80°C for further analysis.

### Cell permeabilization

Digitonin was used to selectively permeabilize the plasma membrane of cells but not the organelle membrane, as previously described [46]. Briefly, about 10^6^ HeLa cells were transfected with the indicated expression vectors. After 48 hours, the cells were trypsinized and collected by centrifugation. Half of the cells were lysed in 500 µl of RIPA buffer supplemented with competent protease-inhibitor cocktail (Roche), and the other half were resuspended in 500 µl of transport buffer (20 mM HEPES, pH 7.4, 110 mM potassium acetate, 2 mM magnesium acetate, 0.5 mM EGTA) supplemented with proteinase inhibitor cocktail. Cell permeabilization was carried out in 0.01% digitonin for 5 minutes on ice. The supernatant and pellet were collected by centrifugation. The cell pellet was resuspended in 500 µl of RIPA buffer. Equivalent volumes of intact cells, permeabilized cells, and the released fraction were analyzed by Western blot.

### Western blot analysis

Cells were lysed in RIPA buffer supplemented with competent protease-inhibitor mixture (Roche) and 50 µg cell lysate was subjected to SDS-PAGE. The immunoblot membranes were probed with primary antibody and the protein signals were detected with the ECL reagent (Roche) or Supersignal chemiluminescence reagent (PIERCE) after reacting with HRP-conjugated secondary antibody. The source and dilution of the primary antibodies were listed below.

Goat anti-GRP78 (C-20) (1∶1000), rabbit anti-HA (1∶1000), goat anti-PERK (1∶1000), rabbit anti-ATF4 (1∶1000), monoclonal mouse anti-CHOP (1∶2000) and rabbit anti-ATF6 (1∶1000) are from Santa Cruz Biotechnology. Rabbit anti-phospho-PERK (1∶1000), rabbit anti-p-eIF2α (1∶1000), rabbit anti-eIF2α (1∶1000) and rabbit anti-cleaved caspase-3 (1∶2000) are from Cell Signaling Biotechnology. Monoclonal mouse anti-β-actin (1∶5000) and monoclonal mouse anti-FLAG (1∶5000) are from Sigma. Monoclonal mouse anti-GRP78 (1∶2000, Cat. No. 610978) and monoclonal mouse anti-caspase-7 antibody (1∶4000) are from BD Pharmingen. Monoclonal mouse anti-P58^IPK^ is a gift from M.G. Katze (University of Washington).

### Confocal immunofluorescence microscopy

The cells were fixed with 4% paraformaldehyde at room temperature for 10 minutes and washed twice with PBS. Immunofluorescence staining was performed as previously described [70]. The primary antibodies used are anti-HA polyclonal antibody and anti-GRP78 (H-129) antibody (1∶500, Santa Cruz Biotechnology), followed by FITC-conjugated secondary antibodies (Vector Labs). The cells were mounted in Vectashield mounting medium (Vector Labs) and visualized on a Perkin-Elmer/Zeiss confocal microscope.

### Co-immunoprecipitation assay

The protocol for co-immunoprecipitation with the anti-FLAG antibody has been described [14].

### Soft agar colongenic survival assay

A 0.3% soft agar solution was prepared by adding one volume of autoclaved 1.5% low melting temperature agarose to four volume of RPMI 1640 medium. HL-60 cells were mixed with the soft agar solution at a density of 300 cells/well in 6-well plates. The plating was performed in triplicate. Ten days later, the colonies with more than 50 cells were counted under inverted microscope. The survival fraction was normalized by dividing the number of surviving colonies of treated groups by that of untreated control groups.

## Supporting Information

Supporting Information S1 Supporting information and figure legends
(0.05 MB DOC)

Figure S1 Discovery of the Alternative Transcript of Grp78. (A) Schematic representation of the intron 1 splicing in mouse Grp78. Arrows labeled as p1 and p2a indicate the primers used in RT-PCR. (B) RT-PCR was performed with cDNA samples from Tg-treated or non-treated NIH3T3 cells by using the primer set p1p2a. To assess the genomic contamination, PCR with the total RNA samples used for reverse transcription was performed. The gel image is shown with inverted background. (C) Alignment of the intron 1 sequence of human and mouse Grp78va. Arrows indicate the suboptimal splicing sites in human and mouse Grp78va. (D) Alignment of the primer p2a flanking sequence and the junction sequence of intron 1 and exon2 in mouse Grp78.
(0.50 MB TIF)

Figure S2 Induction of Grp78va Transcript by Tunicamycin and L-azetidine 2-carboxylic Acid. HeLa cells were either non-treated (−) or treated with 1.5 ug/ml tunicamycin (Tun) or 5 mM L-azetidine 2-carboxylic acid (AzC) for 16 h. Total RNA was subjected to RT-PCR with the specific primers (p1/3 and p4/5) for the human homologue of mGrp78va shown in Figure 1A. b-actin levels served as control.
(0.18 MB TIF)

Figure S3 Bioinformatic Analysis of Intron 1 Retention Alternative Splicing of Human Grp78 by Using a Microarray Database. Analysis of the microarray data was described in the Supporting Information Materials and Methods. The values of X-axis show the difference between the intensities of exon 1/2 and exon 2/3 probes in each tissue sample and the numbers plotted vertically correspond to human tissues or cancer cell lines included in the microarray database.
(0.65 MB TIF)

Figure S4 Detection of GRP78 but not GRP78va by an Anti-N Terminus GRP78 Antibody. HeLa cells were transiently transfected with pcDNA3 (lane 1) or pcDNA/GRP78va-sm (lane 2) for 48 h. The cell lysates were analyzed by Western blots. Left panel shows immunoblot with anti-N-terminus GRP78 antibody (N-20 from Santa Cruz Biotechnology); right panel shows immunoblot with anti-C-terminus GRP78 antibody (C 20 from Santa Cruz Biotechnology). In the left panel, the asterisk (*) denotes a non-specific protein band that immunoreacts with the N-20 antibody.
(0.23 MB TIF)

Figure S5 GRP78va is the Protein Product of the Grp78va Transcript. (A) Schematic diagram of Grp78va and canonical Grp78 mRNA showing the target (red arrow) of Grp78va-specific siRNA (siGrp78va) in intron 1 retained in Grp78va mRNA. (B) HeLa cells were transfected with siCtrl or siGrp78va for 72 h. The Grp78va and total Grp78 mRNA levels were evaluated by quantitative real-time PCR. The results were summarized and plotted with standard deviations. (C) HeLa cells were transfected with siCtrl or siGrp78va for 72 h. Endogenous GRP78va and canonical GRP78 were detected by Western blots using anti-GRP78 monoclonal antibody with b-actin as loading control. For the abundant canonical GRP78, a light exposure is shown. (D) The experiments described in (C) were repeated three times. The GRP78va and GRP78 protein levels were quantitated and normalized to b-actin level. The results were summarized and plotted with standard deviations.
(0.53 MB TIF)

Figure S6 Dual Localization of P58IPK in the ER and the Cytosol. HeLa cells transiently transfected with pcDNA/P58IPK-FLAG were fixed in methanol and stained with anti-FLAG monoclonal antibody (1∶1000, Sigma) for detection of P58IPK (red), followed by anti-PDI polyclonal antibody (1∶500, Santa Cruz Biotechnology) as the ER marker (green), and then DAPI to indicate the nucleus (blue). The cells were subjected to confocal microscopy. The individual images and the merged image are shown.
(2.10 MB TIF)

Figure S7 GRP78va Promotes HeLa Cell Survival During ER Stress. (A) HeLa cells transfected with siCtrl or siGrp78va were exposed to Tg (1 uM) for the indicated hours and then re-plated in fresh medium for colongenic survival assay (see Supporting Information Materials and Methods). After 10–14 days, the colonies were counted and the survival fractions were plotted against the time of treatment as indicated. (B) HeLa cells stably overexpressing GRP78va (Grp78va) or control cells (vector) were treated with Tg (1 uM) for the indicated hours and then subjected to colongenic survival assay.
(0.32 MB TIF)

Figure S8 GRP78va is Stabilized by Proteasome Inhibitor (A) Proteasome inhibitor stabilizes overexpressed HA-GRP78va. 293T cells transiently transfected with pcDNA/HA-Grp78va were either non-treated (−) or treated (+) with MG115 (2 h) or Tg (16 h) as indicated and subjected to Western blots. The HA-GRP78va levels normalized to b-actin are indicated below. (B) GRP78va has a short half-life. 293T cells were transfected with pcDNA/HA-GRP78va and after 24 h, the cells were seeded onto the 6-well plates. Next day, the cells were treated with cycloheximide (CHX) for the time (in hours) indicated on top. The cell lysates were collected and analyzed by Western blot using antibodies against GRP78 (C-20) and b-actin. The positions of GRP78 and HA-GRP78va are indicated. The HA-GRP78va levels normalized to b-actin are indicated below. (C) Alignment of human and mouse GRP78va with human GRP78 protein sequence. The mouse GRP78va amino acids divergent from human GRP78 and GRP78va are highlighted in red. The PEST-rich region is boxed, and the enrichment of proline (P), glutamic acid (E), serine (S), and threonine (T) residues are bolded.
(0.67 MB TIF)

Table S1 Summary of alternative splicing by intron retention from Grp78va gene.
(0.53 MB TIF)
