# Supplementary material for: Regulation of PERK Signaling and Leukemic Cell Survival by a Novel Cytosolic Isoform of the UPR Regulator GRP78/BiP
Source: PLoS One. 2009 Aug 31;4(8):e6868. doi: 10.1371/journal.pone.0006868 (PMC2729930; doi:10.1371/journal.pone.0006868)
Supplement: Supporting Information S1 — Supporting information and figure legends (0.05 MB DOC) [file pone.0006868.s001.doc]

**Supporting Information**

**Primers**

A. Primers for RT-PCR

Mouse *Grp78* and *Grp78va*:

p1: 5'-GCTCCGAGTCTGCTTCGTGTCT-3’

p2a: 5’-GAACACACCGACGCAGGA-3’

p2: 5’-TGGACGTGAGTTGGTTCTTG-3’

p3: 5’-CAGCACCTCACTTTCTCACGCT-3’

Human *Grp78* and *Grp78va*:

p1: 5’-CAGCACAGACAGATTGACCTAT-3’

p3: 5’-GACATCAGCACCGCACTTCTCA-3’

p4: 5’-GGTGCTGATGTCCCTCTGTC-3’

p5: 5’-CCTAACAAAAGTTCCTGAGTCCA-3’

Mouse β*-actin*:

Forward: 5’-GACGGCCAGGTCATCACTAT-3’

Reverse: 5’-GTACTTGCGCTCAGGAGGAG-3’

Human β*-actin*:

Forward: 5’-TCGTGCGTGACATTAAGGAG-3’

Reverse: 5’-AGCACTGTGTTGGCGTACAG-3’

Human *Xbp1*:

Forward: 5’-CTGGAACAGCAAGTGGTAGA-3’

Reverse: 5’-CTGGGTCCTTCTGGGTAGAC-3’

B. Primers for quantitative real-time PCR

*Grp78* forward: 5’-CGACCTGGGGACCACCTACT-3’

*Grp78va* forward: 5’-GGTGCTGATGTCCCTCTGTC-3’

Reverse: 5’-TTGGAGGTGAGCTGGTTCTT-3’

*GAPDH* forward: 5’-TCTGGTAAAGTGGATATTGTTG-3’

*GAPDH* reverse: 5’-GATGGTGATGGGATTTCC-3’

C. Primers used in RT-PCR with leukemia patient samples

*Grp78va* forward: 5’-GGTGCTGATGTCCCTCTGTC-3’

Reverse: 5-GCCACAACTTCGAAGACACCA-3’

D. Primers for plasmid construction

For site-directed mutagenesis:

pcDNA/Grp78va-sm:

5’-CCACCTACTCCTGCTAGCTGGGATTGCGGATGC-3’

5’-GCATCCGCAATCCCAGCTAGCAGGAGTAGGTGG-3’

pcDNA/HA-Grp78va-G80D:

5’-GTGTTTGACCTGGGTGACGGAACCTTCGATG-3’

5’-CATCGAAGGTTCCGTCACCCAGGTCAAACAC-3’

For generation of pcDNA/HA-Grp78va:

Forward: 5’-CGCGGATCCATGGTTCTCACTAAAATGA-3’

Reverse: 5’-CCGCTCGAGCCTAACAAAAGTTCCTGAGTCCA-3’

For generation of pcDNA/FLAG-P58^IPK^:

Forward: 5’-CGCGGATCCTTGGTGGCCCCCGGCT-3’

Reverse: 5’-CCGCTCGAGCAGTTGGTTTAATTGAAGTGGAA-3’

For generation of pcDNA/ P58^IPK^-FLAG:

Forward: 5’-CGCGGATCCATGGTGGCCCCCGGCT-3’

Reverse: 5’-CCGCTCGAGTTACTTGTCATCGTCGTCCTTGTAGTCATTGAAGTGGAA-3’ (Broken underline indicates FLAG coding sequence)

**siRNA**

*Grp78va* siRNA (siGrp78va):

sense: 5'-AAGUGCGGUGCUGAUGUCCCU-3';

antisense: 5'-AGGGACAUCAGCACCGCACUU-3'.

Control siRNA (siCtrl):

sense: 5'-AAGGAGACGUAUAGCAACGGU-3';

antisense: 5'-ACCGUUGCUAUACGUCUCCUU-3'.

**Bioinformatic Analysis**

Large-scale microarrays have been developed for analyzing alternative splicing. After search of NCBI/GEO profiles for human *Grp78* (Hspa5, NM_005347), the expression data from a microarray analysis (accession number GDS831) of alternative pre-mRNA splicing in various human tissues and cell lines was downloaded and applied to further analysis. This array used 36-mer probes positioned at every consecutive exon-exon junction of more than 10,000 multi-exon genes to monitor splice variants and tissue distributions for thousands of alternative splicing events in 52 human tissues and cell lines. The data was calculated by the natural log intensity of each junction probe in one tissue. To normalize the data, the average of values from all seven junction probes and the average of values of one probe in all 52 tissues were subtracted from each value. To predict the intron 1 retention of *Grp78*, the values of exon 1/2 and exon 2/3 were compared by subtracting exon 2/3 intensity from exon 1/2 intensity and the data was ordered and plotted. The smallest value indicated the most significantly down-regulated signal from exon 1/2 junction probe and predicted the highest probability of intron 1 retention.

**cDNA Cloning and Plasmid Construction**

In the first round of the bridging-based PCR, the 5’-part of *Grp78va* was amplified with primers 5’-CGCGGATCCCAGCACAGACAGATTGACCTAT-3’ and p3, and the 3’-part was obtained with primers p4 and 5’-CCGCTCGAGCCTAACAAAAGTTCCT GAGTCCA-3’. Primer p3 and p4 have 12 nucleotides complementary in the sequence of intron 1. The first round PCR products were gel purified and quantified. Fifty ng of left and right overlapping fragments were amplified in standard PCR solution for five rounds of amplification without primers and then 5’-part forward and 3’-part reverse primers were added before PCR for another 30 cycles. The purified PCR products were digested with BamH I and Xho I, and cloned into pcDNA3 vector to generate the *Grp78va* expression plasmid pcDNA-Grp78va. To further generate the splicing donor site mutation of intron 1 in pcDNA-Grp78va, the QuickChange site-directed mutagenesis method (Stratagene) was performed according to manufacturer’s protocol and the mutant plasmid was designated pcDNA-Grp78va-sm. The PCR-amplified *Grp78va* coding sequence was digested by BamH I/Xho I and inserted into the same sites of pcDNA3-HA vector to generate HA-Grp78va expression vector pcDNA/HA-Grp78va. Site-directed mutagenesis was performed on pcDNA/HA-Grp78va to generate the expression vector bearing the ATP binding mutation (G80D). The coding sequence of P58^IPK^ was amplified and cloned into BamH I and Xho I sites of pcDNA3-FLAG vector to generate pcDNA/FLAG-P58^IPK^. For construction of P58^IPK^ expression vector with carboxy-terminal FLAG tag, the FLAG sequence was added by PCR and the fragments were cloned into pcDNA3 to generate pcDNA/P58^IPK^-FLAG. The primers used for plasmid construction were summarized in Primers of the Supporting Information.

**Colongenic Survival Assay for HeLa Cells**

The cells plated into 12-well plates were treated with Tg for indicated hours or left untreated. After treatment, cells were allowed to recover for 2 h and then seeded into 6-cm-diameter dishes at a density of 300 cells/dish. Plating was performed in triplicate. After growing in fresh medium for 10-14 days, the colonies were washed with ice-cold PBS, fixed with methanol and stained with 10% Giemsa staining solution. The survival fraction was normalized by dividing the number of surviving colonies of treated samples by that of untreated control samples. The experiments were repeated three times.

**Cycloheximide Chase Assay**

293T cells were transfected with pcDNA/HA-GRP78va. After 24 h, the transfected cells were seeded onto 6-well plate, and the next day treated with 100 μg/ml cycloheximide for 0, 1, 2, 4 and 6 h. The cell lysates were collected and protein levels analyzed by Western blots. The blots were quantified by Quantity One software.

**Figure S1.** Discovery of the Alternative Transcript of *Grp78*

(A) Schematic representation of the intron 1 splicing in mouse *Grp78*. Arrows labeled as p1 and p2a indicate the primers used in RT-PCR.

(B) RT-PCR was performed with cDNA samples from Tg-treated or non-treated NIH3T3 cells by using the primer set p1p2a. To assess the genomic contamination, PCR with the total RNA samples used for reverse transcription was performed. The gel image is shown with inverted background.

(C) Alignment of the intron 1 sequence of human and mouse *Grp78va*. Arrows indicate the suboptimal splicing sites in human and mouse *Grp78va*.

(D) Alignment of the primer p2a flanking sequence and the junction sequence of intron 1 and exon2 in mouse *Grp78*.

**Figure S2.** Induction of *Grp78va* Transcript by Tunicamycin and L-azetidine 2-carboxylic Acid

HeLa cells were either non-treated (-) or treated with 1.5 μg/ml tunicamycin (Tun) or 5 mM L-azetidine 2-carboxylic acid (AzC) for 16 h. Total RNA was subjected to RT-PCR with the specific primers (p1/3 and p4/5) for the human homologue of m*Grp78va* shown in Figure 1A. β-actin levels served as control.

**Figure S3.** Bioinformatic Analysis of Intron 1 Retention Alternative Splicing of Human *Grp78* by Using a Microarray Database

Analysis of the microarray data was described in the Supporting Information Materials and Methods. The values of X-axis show the difference between the intensities of exon 1/2 and exon 2/3 probes in each tissue sample and the numbers plotted vertically correspond to human tissues or cancer cell lines included in the microarray database.

**Figure S4.** Detection of GRP78 but not GRP78va by an Anti-N Terminus GRP78 Antibody

HeLa cells were transiently transfected with pcDNA3 (lane 1) or pcDNA/GRP78va-sm (lane 2) for 48 h. The cell lysates were analyzed by Western blots. Left panel shows immunoblot with anti-N-terminus GRP78 antibody (N-20 from Santa Cruz Biotechnology); right panel shows immunoblot with anti-C-terminus GRP78 antibody (C‑20 from Santa Cruz Biotechnology). In the left panel, the asterisk (*) denotes a non-specific protein band that immunoreacts with the N-20 antibody.

**Figure S5.** GRP78va is the Protein Product of the *Grp78va* Transcript

(A) Schematic diagram of *Grp78va* and canonical *Grp78* mRNA showing the target (red arrow) of *Grp78va*-specific siRNA (siGrp78va) in intron 1 retained in *Grp78va* mRNA.

(B) HeLa cells were transfected with siCtrl or siGrp78va for 72 h. The Grp78va and total Grp78 mRNA levels were evaluated by quantitative real-time PCR. The results were summarized and plotted with standard deviations.

(C) HeLa cells were transfected with siCtrl or siGrp78va for 72 h. Endogenous GRP78va and canonical GRP78 were detected by Western blots using anti-GRP78 monoclonal antibody with β-actin as loading control. For the abundant canonical GRP78, a light exposure is shown.

(D) The experiments described in (C) were repeated three times. The GRP78va and GRP78 protein levels were quantitated and normalized to β-actin level. The results were summarized and plotted with standard deviations.

**Figure S6.** Dual Localization of P58^IPK^ in the ER and the Cytosol

HeLa cells transiently transfected with pcDNA/P58^IPK^-FLAG were fixed in methanol and stained with anti-FLAG monoclonal antibody (1:1000, Sigma) for detection of P58^IPK^ (red), followed by anti-PDI polyclonal antibody (1:500, Santa Cruz Biotechnology) as the ER marker (green), and then DAPI to indicate the nucleus (blue). The cells were subjected to confocal microscopy. The individual images and the merged image are shown.

**Figure S7.** GRP78va Promotes HeLa Cell Survival During ER Stress

(A) HeLa cells transfected with siCtrl or siGrp78va were exposed to Tg (1 μM) for the indicated hours and then re-plated in fresh medium for colongenic survival assay (see Supporting Information Materials and Methods). After 10-14 days, the colonies were counted and the survival fractions were plotted against the time of treatment as indicated.

(B) HeLa cells stably overexpressing GRP78va (Grp78va) or control cells (vector) were treated with Tg (1 μM) for the indicated hours and then subjected to colongenic survival assay.

**Figure S8.** GRP78va is Stabilized by Proteasome Inhibitor

(A) Proteasome inhibitor stabilizes overexpressed HA-GRP78va. 293T cells transiently transfected with pcDNA/HA-Grp78va were either non-treated (-) or treated (+) with MG115 (2 h) or Tg (16 h) as indicated and subjected to Western blots. The HA-GRP78va levels normalized to β-actin are indicated below.

(B) GRP78va has a short half-life. 293T cells were transfected with pcDNA/HA-GRP78va and after 24 h, the cells were seeded onto the 6-well plates. Next day, the cells were treated with cycloheximide (CHX) for the time (in hours) indicated on top. The cell lysates were collected and analyzed by Western blot using antibodies against GRP78 (C-20) and β-actin. The positions of GRP78 and HA-GRP78va are indicated. The HA-GRP78va levels normalized to β-actin are indicated below.

(C) Alignment of human and mouse GRP78va with human GRP78 protein sequence. The mouse GRP78va amino acids divergent from human GRP78 and GRP78va are highlighted in red. The PEST-rich region is boxed, and the enrichment of proline (P), glutamic acid (E), serine (S), and threonine (T) residues are bolded.

**Table S1.** Summary of alternative splicing by intron retention from *Grp78va* gene.
