# Supplementary figures and images for: Regulation of PERK Signaling and Leukemic Cell Survival by a Novel Cytosolic Isoform of the UPR Regulator GRP78/BiP
Source: PLoS One. 2009 Aug 31;4(8):e6868. doi: 10.1371/journal.pone.0006868 (PMC2729930; doi:10.1371/journal.pone.0006868)

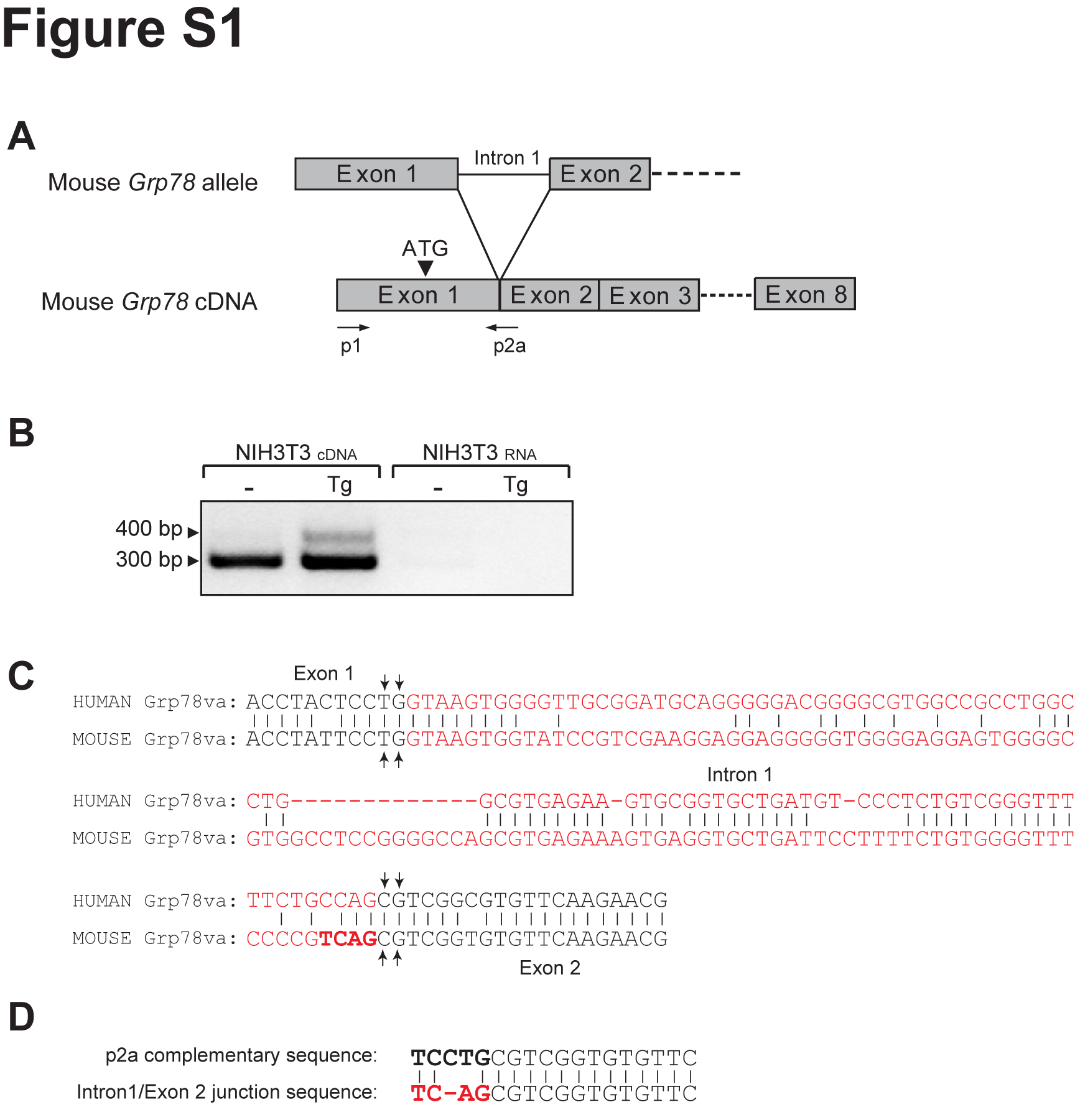

Supplement: Figure S1 — Discovery of the Alternative Transcript of Grp78. (A) Schematic representation of the intron 1 splicing in mouse Grp78. Arrows labeled as p1 and p2a indicate the primers used in RT-PCR. (B) RT-PCR was performed with cDNA samples from Tg-treated or non-treated NIH3T3 cells by using the primer set p1p2a. To assess the genomic contamination, PCR with the total RNA samples used for reverse transcription was performed. The gel image is shown with inverted background. (C) Alignment of the intron 1 sequence of human and mouse Grp78va. Arrows indicate the suboptimal splicing sites in human and mouse Grp78va. (D) Alignment of the primer p2a flanking sequence and the junction sequence of intron 1 and exon2 in mouse Grp78. (0.50 MB TIF) [file pone.0006868.s002.tif]

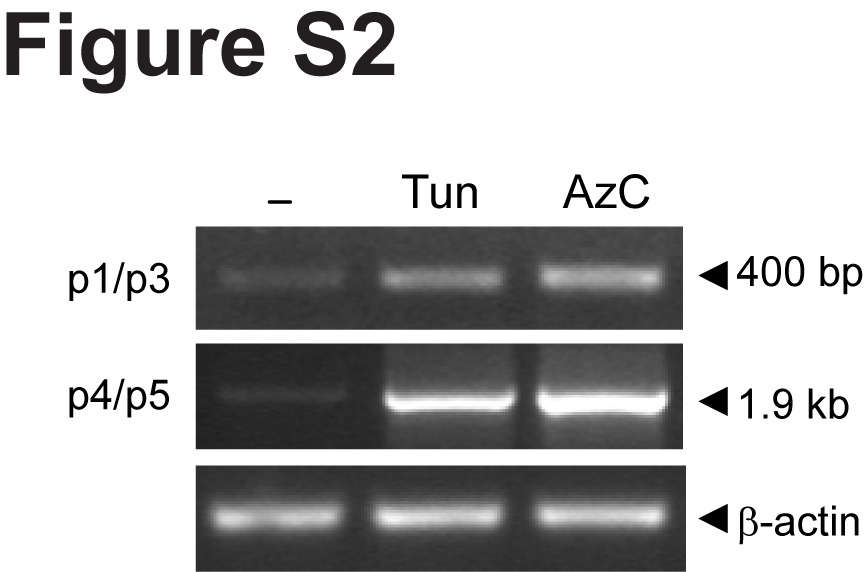

Supplement: Figure S2 — Induction of Grp78va Transcript by Tunicamycin and L-azetidine 2-carboxylic Acid. HeLa cells were either non-treated (−) or treated with 1.5 ug/ml tunicamycin (Tun) or 5 mM L-azetidine 2-carboxylic acid (AzC) for 16 h. Total RNA was subjected to RT-PCR with the specific primers (p1/3 and p4/5) for the human homologue of mGrp78va shown in Figure 1A. b-actin levels served as control. (0.18 MB TIF) [file pone.0006868.s003.tif]

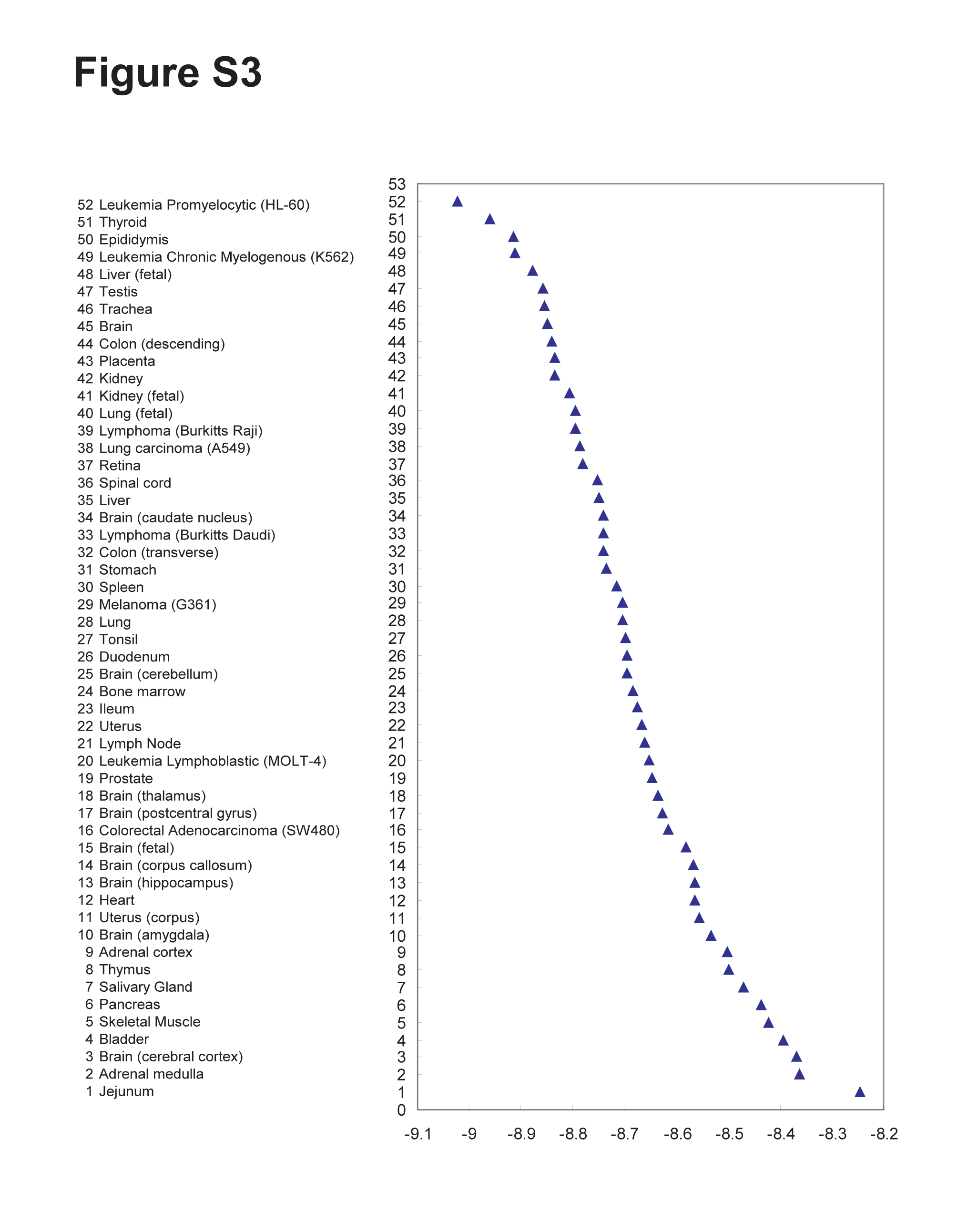

Supplement: Figure S3 — Bioinformatic Analysis of Intron 1 Retention Alternative Splicing of Human Grp78 by Using a Microarray Database. Analysis of the microarray data was described in the Supporting Information Materials and Methods. The values of X-axis show the difference between the intensities of exon 1/2 and exon 2/3 probes in each tissue sample and the numbers plotted vertically correspond to human tissues or cancer cell lines included in the microarray database. (0.65 MB TIF) [file pone.0006868.s004.tif]

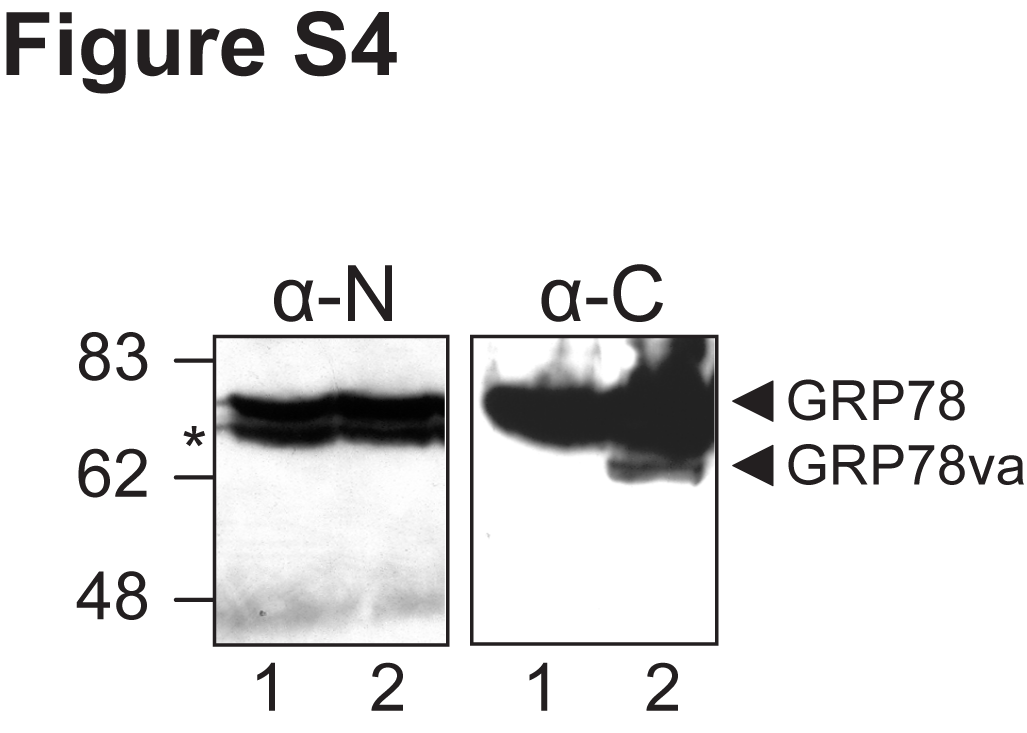

Supplement: Figure S4 — Detection of GRP78 but not GRP78va by an Anti-N Terminus GRP78 Antibody. HeLa cells were transiently transfected with pcDNA3 (lane 1) or pcDNA/GRP78va-sm (lane 2) for 48 h. The cell lysates were analyzed by Western blots. Left panel shows immunoblot with anti-N-terminus GRP78 antibody (N-20 from Santa Cruz Biotechnology); right panel shows immunoblot with anti-C-terminus GRP78 antibody (C 20 from Santa Cruz Biotechnology). In the left panel, the asterisk (*) denotes a non-specific protein band that immunoreacts with the N-20 antibody. (0.23 MB TIF) [file pone.0006868.s005.tif]

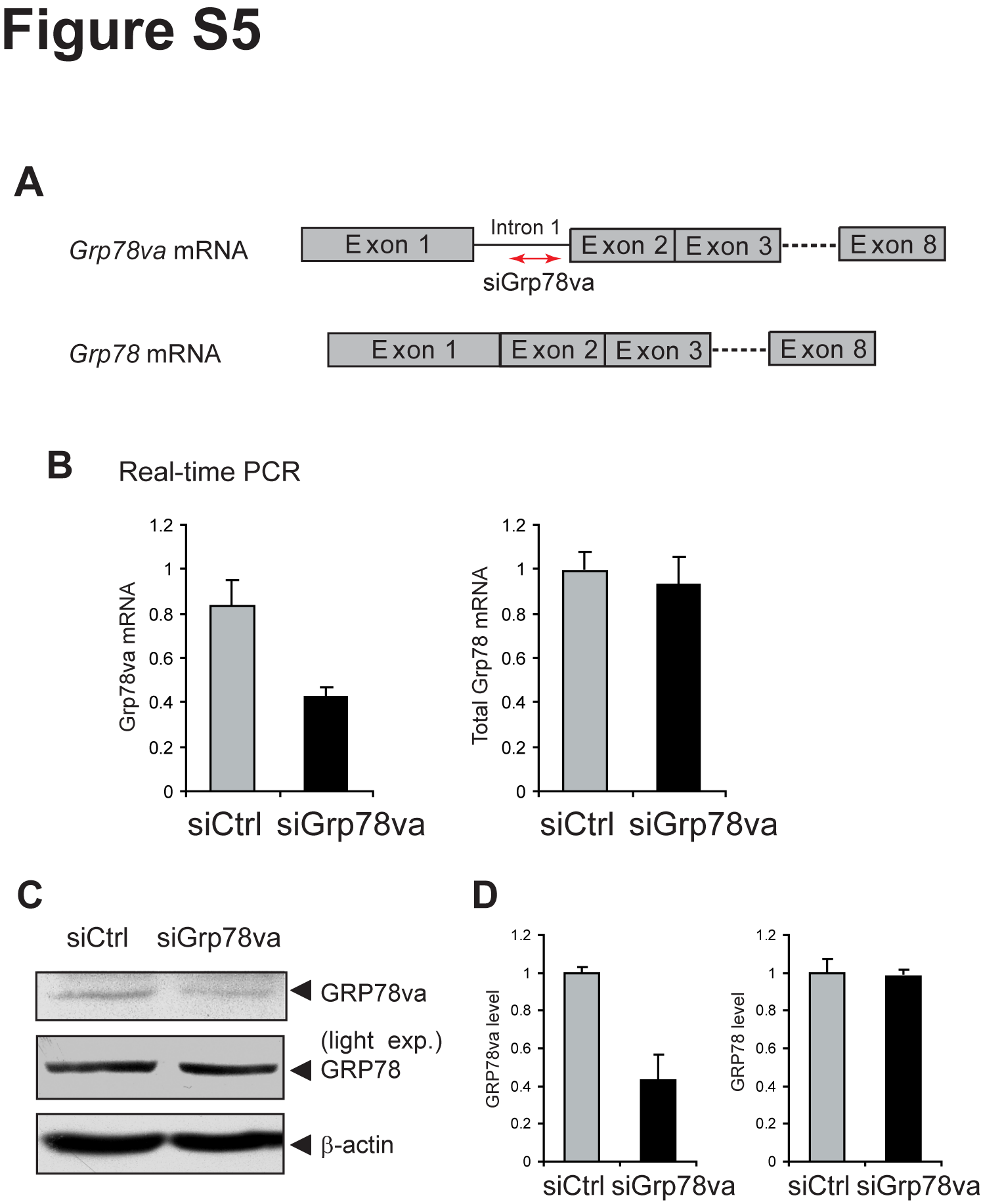

Supplement: Figure S5 — GRP78va is the Protein Product of the Grp78va Transcript. (A) Schematic diagram of Grp78va and canonical Grp78 mRNA showing the target (red arrow) of Grp78va-specific siRNA (siGrp78va) in intron 1 retained in Grp78va mRNA. (B) HeLa cells were transfected with siCtrl or siGrp78va for 72 h. The Grp78va and total Grp78 mRNA levels were evaluated by quantitative real-time PCR. The results were summarized and plotted with standard deviations. (C) HeLa cells were transfected with siCtrl or siGrp78va for 72 h. Endogenous GRP78va and canonical GRP78 were detected by Western blots using anti-GRP78 monoclonal antibody with b-actin as loading control. For the abundant canonical GRP78, a light exposure is shown. (D) The experiments described in (C) were repeated three times. The GRP78va and GRP78 protein levels were quantitated and normalized to b-actin level. The results were summarized and plotted with standard deviations. (0.53 MB TIF) [file pone.0006868.s006.tif]

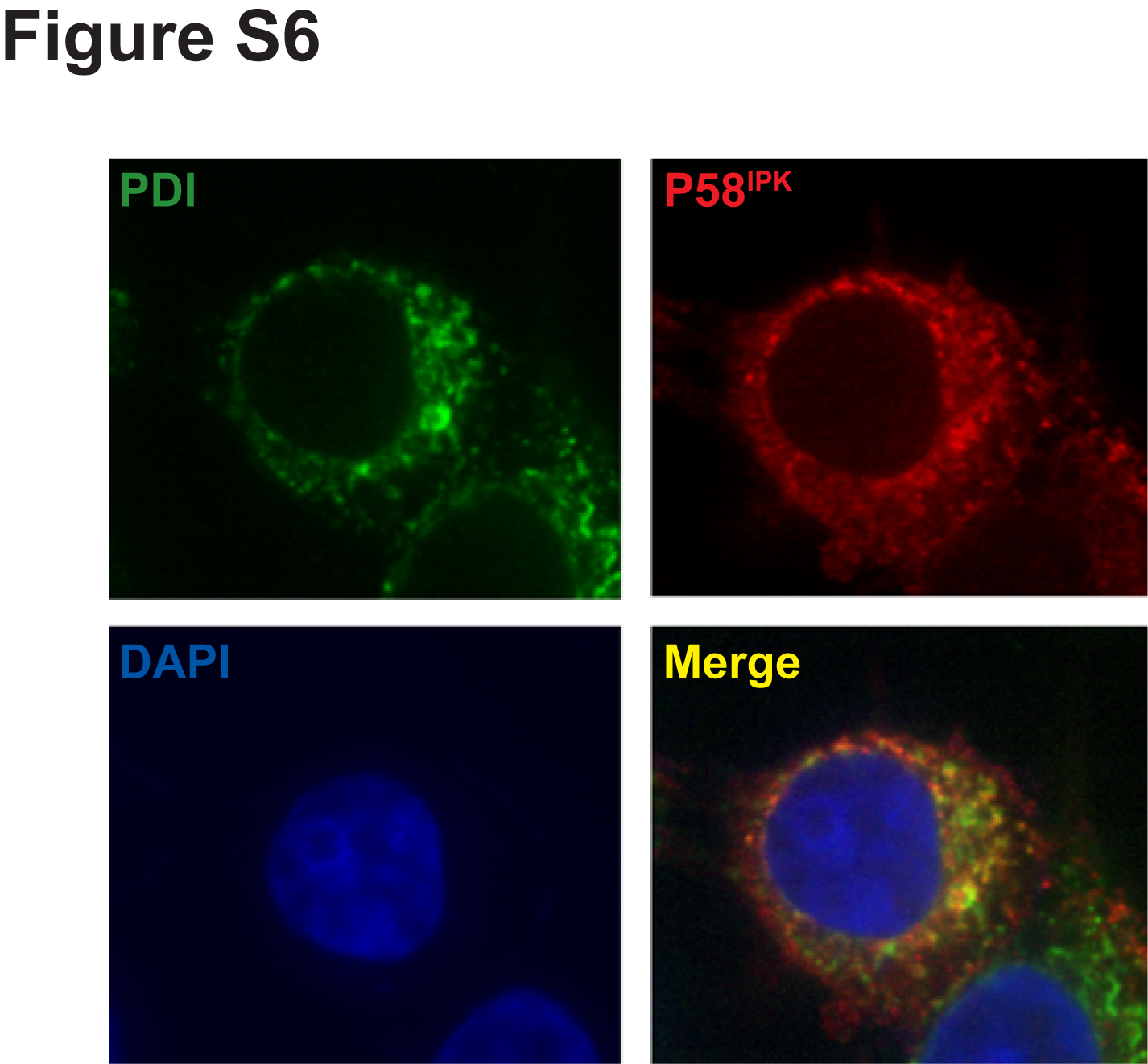

Supplement: Figure S6 — Dual Localization of P58IPK in the ER and the Cytosol. HeLa cells transiently transfected with pcDNA/P58IPK-FLAG were fixed in methanol and stained with anti-FLAG monoclonal antibody (1∶1000, Sigma) for detection of P58IPK (red), followed by anti-PDI polyclonal antibody (1∶500, Santa Cruz Biotechnology) as the ER marker (green), and then DAPI to indicate the nucleus (blue). The cells were subjected to confocal microscopy. The individual images and the merged image are shown. (2.10 MB TIF) [file pone.0006868.s007.tif]

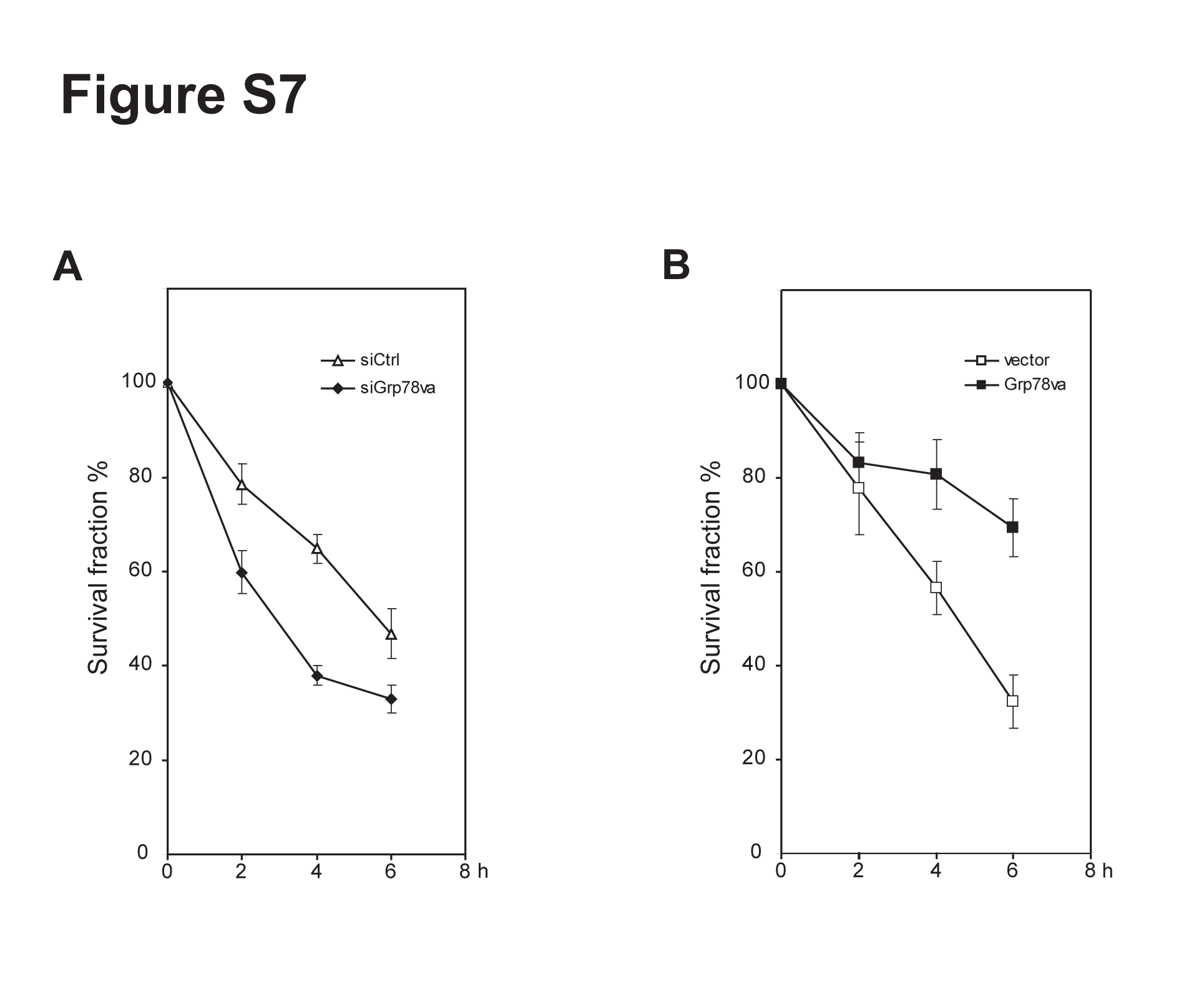

Supplement: Figure S7 — GRP78va Promotes HeLa Cell Survival During ER Stress. (A) HeLa cells transfected with siCtrl or siGrp78va were exposed to Tg (1 uM) for the indicated hours and then re-plated in fresh medium for colongenic survival assay (see Supporting Information Materials and Methods). After 10–14 days, the colonies were counted and the survival fractions were plotted against the time of treatment as indicated. (B) HeLa cells stably overexpressing GRP78va (Grp78va) or control cells (vector) were treated with Tg (1 uM) for the indicated hours and then subjected to colongenic survival assay. (0.32 MB TIF) [file pone.0006868.s008.tif]

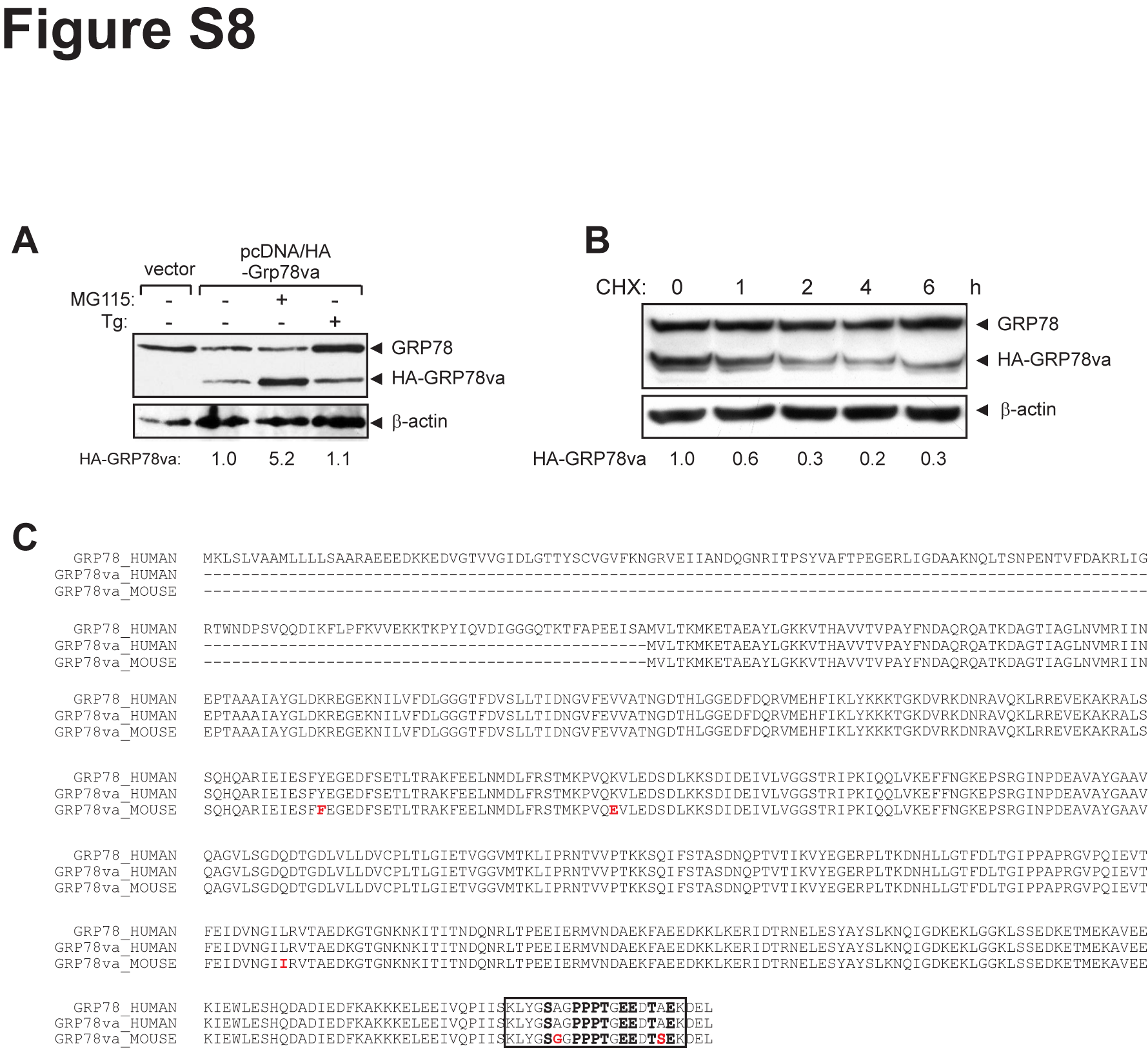

Supplement: Figure S8 — GRP78va is Stabilized by Proteasome Inhibitor (A) Proteasome inhibitor stabilizes overexpressed HA-GRP78va. 293T cells transiently transfected with pcDNA/HA-Grp78va were either non-treated (−) or treated (+) with MG115 (2 h) or Tg (16 h) as indicated and subjected to Western blots. The HA-GRP78va levels normalized to b-actin are indicated below. (B) GRP78va has a short half-life. 293T cells were transfected with pcDNA/HA-GRP78va and after 24 h, the cells were seeded onto the 6-well plates. Next day, the cells were treated with cycloheximide (CHX) for the time (in hours) indicated on top. The cell lysates were collected and analyzed by Western blot using antibodies against GRP78 (C-20) and b-actin. The positions of GRP78 and HA-GRP78va are indicated. The HA-GRP78va levels normalized to b-actin are indicated below. (C) Alignment of human and mouse GRP78va with human GRP78 protein sequence. The mouse GRP78va amino acids divergent from human GRP78 and GRP78va are highlighted in red. The PEST-rich region is boxed, and the enrichment of proline (P), glutamic acid (E), serine (S), and threonine (T) residues are bolded. (0.67 MB TIF) [file pone.0006868.s009.tif]

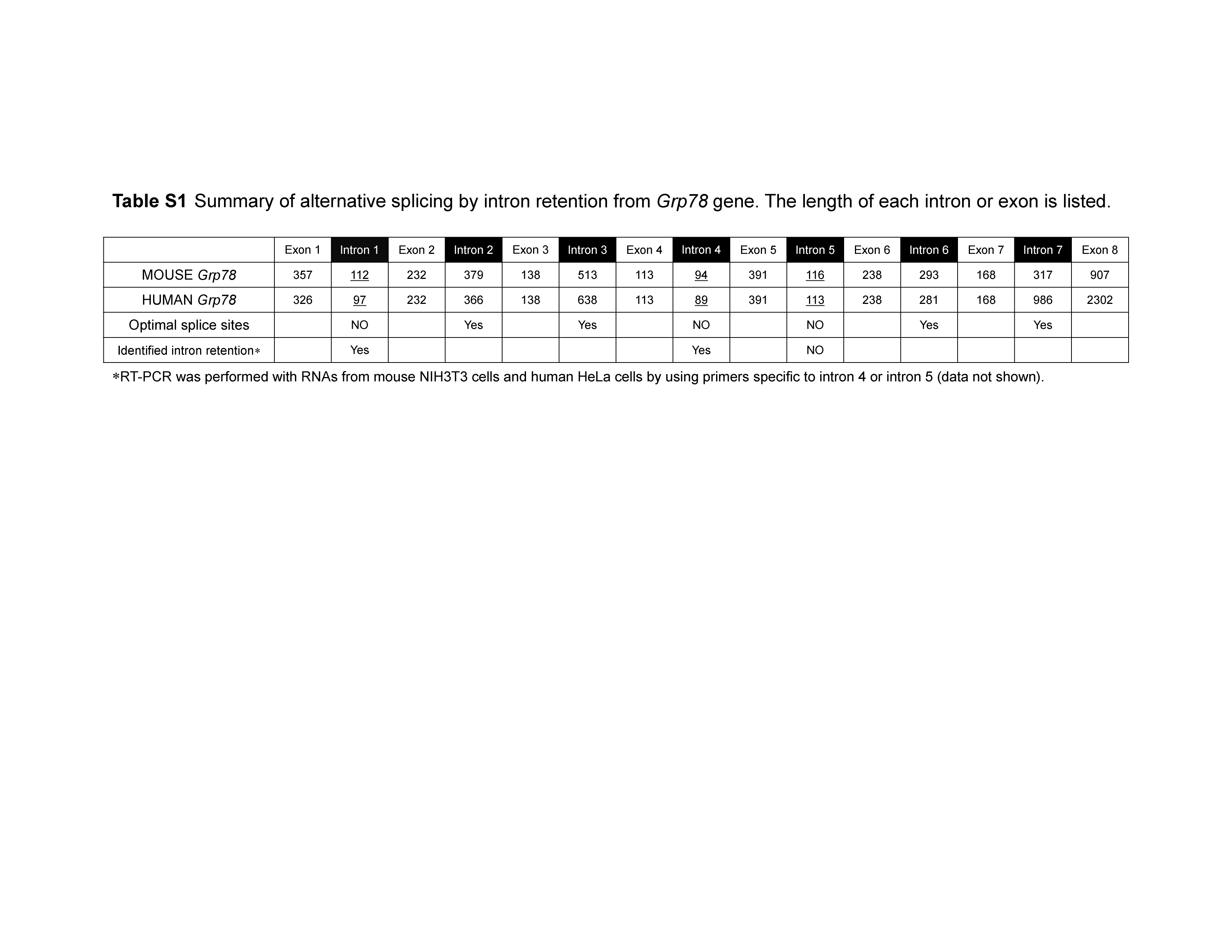

Supplement: Table S1 — Summary of alternative splicing by intron retention from Grp78va gene. (0.53 MB TIF) [file pone.0006868.s010.tif]
